# Structures and Magnetic Ordering in Layered Cr Oxide Arsenides Sr_2_CrO_2_Cr_2_OAs_2_ and Sr_2_CrO_3_CrAs

**DOI:** 10.1021/acs.inorgchem.2c01773

**Published:** 2022-07-27

**Authors:** Bradley C. Sheath, Xiaoyu Xu, Pascal Manuel, Joke Hadermann, Maria Batuk, John O’Sullivan, Ruy S. Bonilla, Simon J. Clarke

**Affiliations:** † Department of Chemistry, University of Oxford, Inorganic Chemistry Laboratory, South Parks Road, Oxford OX1 3QR, United Kingdom; ‡ ISIS Facility, STFC Rutherford Appleton Laboratory, Harwell Oxford, Didcot OX11 0QX, United Kingdom; § Electron Microscopy for Materials Science (EMAT), University of Antwerp, Groenenborgerlaan 171, B-2020 Antwerp, Belgium; ∥ Department of Materials, University of Oxford, Engineering and Technology Building, Parks Road, Oxford OX1 3PH, United Kingdom

## Abstract

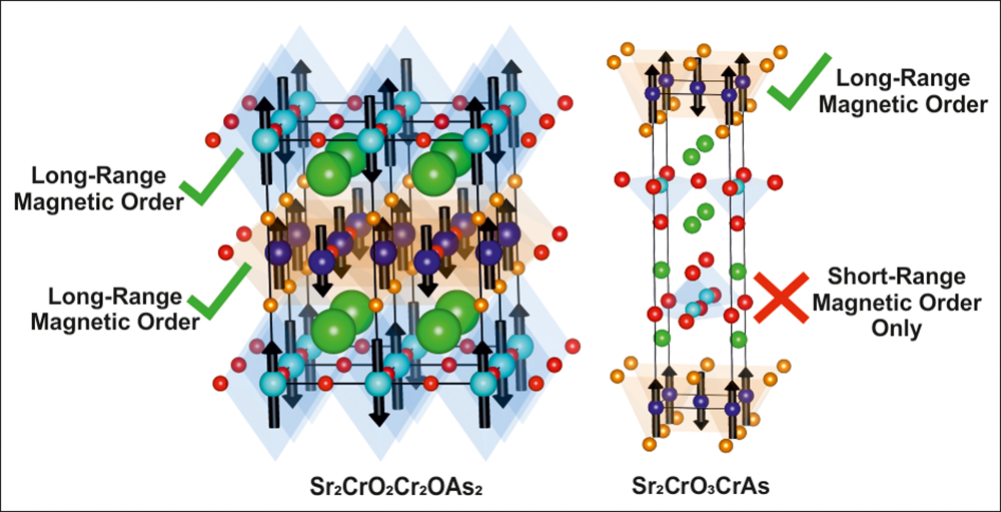

Two novel chromium oxide arsenide materials have been synthesized, Sr_2_CrO_2_Cr_2_OAs_2_ (i.e., Sr_2_Cr_3_As_2_O_3_) and Sr_2_CrO_3_CrAs (i.e., Sr_2_Cr_2_AsO_3_), both of which contain chromium ions in two distinct layers. Sr_2_CrO_2_Cr_2_OAs_2_ was targeted following electron microscopy measurements on a related phase. It crystallizes in the space group *P*4/*mmm* and accommodates distorted CrO_4_As_2_ octahedra containing Cr^2+^ and distorted CrO_2_As_4_ octahedra containing Cr^3+^. In contrast, Sr_2_CrO_3_CrAs incorporates Cr^3+^ in CrO_5_ square-pyramidal coordination in [Sr_2_CrO_3_]^+^ layers and Cr^2+^ ions in CrAs_4_ tetrahedra in [CrAs]^−^ layers and crystallizes in the space group *P*4/*nmm*. Powder neutron diffraction data reveal antiferromagnetic ordering in both compounds. In Sr_2_CrO_3_CrAs the Cr^2+^ moments in the [CrAs]^−^ layers exhibit long-range ordering, while the Cr^3+^ moments in the [Sr_2_CrO_3_]^+^ layers only exhibit short-range ordering. However, in Sr_2_CrO_2_Cr_2_OAs_2_, both the Cr^2+^ moments in the CrO_4_As_2_ environments and the Cr^3+^ moments in the CrO_2_As_4_ polyhedra are long-range-ordered below 530(10) K. Above this temperature, only the Cr^3+^ moments are ordered with a Néel temperature slightly in excess of 600 K. A subtle structural change is evident in Sr_2_CrO_2_Cr_2_OAs_2_ below the magnetic ordering transitions.

## Abstract 2

Sr_2_CrO_2_Cr_2_OAs_2_ and Sr_2_CrO_3_CrAs are both mixed-anion materials containing chromium ions in two unique layers. In Sr_2_CrO_2_Cr_2_OAs_2_, Cr^3+^ ions in CrO_2_As_4_ environments order antiferromagnetically at around 600 K and Cr^2+^ ions in CrO_4_As_2_ environments also order antiferromagnetically at a lower temperature of 530(10) K. In contrast, only the Cr^2+^ moments in the [CrAs]^−^ layers exhibit long-range ordering in Sr_2_CrO_3_CrAs as the Cr^3+^ moments in the [Sr_2_CrO_3_]^+^ layers only exhibit short-range ordering.

## Introduction

With increasing numbers of investigations into crystalline materials containing more than one anion over the last few decades, new phases are being discovered, and along with them come unexplored structures and properties. Research into these mixed-anion solids has been driven in recent years by the search for compounds that are high-temperature superconductors,^1,2^ thermoelectrics,^3^ fast-ion conductors,^4^ and transparent conductors.^5^ Another feature of interest is the nature of any long-range magnetic order present. In mixed-anion compounds such as the oxide arsenides described here and a related series of oxide chalcogenides,^6−10^ the tendency of the different anions to segregate into different layers, due to their different sizes and electronegativities, can lead to the formation of multiple transition metal sublattices within the same structure. If these transition metals, situated in different coordination environments, are magnetic ions, then there is the prospect of novel and complex long-range magnetic order. A plethora of oxypnictide and oxychalcogenide compositions that contain very similar types of layers to the two title compounds have been investigated over the last decade, including Ba_2_Ti_2_OAs_2_Cr_2_As_2_^11^ with TiO_2_As_4_ octahedra and CrAs_4_ tetrahedra, BaTi_2_OAs_2_^12^ with TiO_2_As_4_ octahedra, and Sr_2_CrO_3_FeAs^13^ with CrO_5_ square-based pyramids.

**Figure 1:**
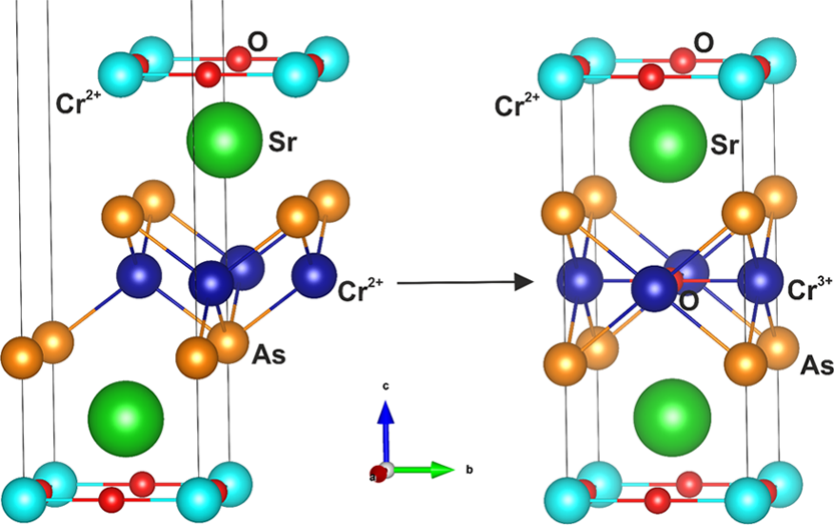
Crystal structures of Sr_2_CrO_2_Cr_2_As_2_ (left)^14,15,21^ and Sr_2_CrO_2_Cr_2_OAs_2_ (right). The structure of Sr_2_CrO_2_Cr_2_OAs_2_ may formally be derived from oxidation of the [Cr_2_As_2_]^2–^ layers in Sr_2_CrO_2_Cr_2_As_2_ and relative shifts of the [Sr_2_CrO_2_]^2+^ slabs.

We previously reported^14^ that the compounds *Ae*_2_CrO_2_Cr_2_As_2_ (*Ae* = Sr, Ba) adopt a structure containing alternating [*Ae*_2_CrO_2_]^2+^ layers (with CrO_2_ square sheets and the Cr^2+^ ions in distended CrO_4_As_2_ octahedra) and anti-PbO [Cr_2_As_2_]^2–^ layers (with edge-sharing CrAs_4_ tetrahedra) as depicted in Figure 1 (left). Cr^2+^ stabilized under the relatively reducing reaction conditions is present on both Cr sublattices. The formula for this and the related compounds reported in this work are written so as to emphasize these different structural slabs. The compound Sr_2_CrO_2_Cr_2_As_2_ is an example of one with complex magnetic order. As described by Liu et al.^15^ and by us (Xu et al.^14^), long-range antiferromagnetic order of the moments on the Cr ions on the [Cr_2_As_2_]^2–^ layer occurs below 590 K, and just below room temperature, the moments on the Cr ions on the oxide layer also start to order antiferromagnetically. The ordering of the oxide layer precipitates a reorientation of the moments in the arsenide layers even though the two Cr sublattices order with different propagation vectors. We concluded from the analysis of high-resolution neutron powder diffraction data that the magnetic structure was slightly incommensurate with the nuclear structure, which presumably enables communication between the two magnetic sublattices. Spin reorientations in related phases have been reported by Lawrence et al.^16^ and by Xu.^17^ We reported^14^ that high-resolution transmission electron microscopy (TEM) measurements performed on Sr_2_CrO_2_Cr_2_As_2_ revealed the presence of stacking faults, which were presumed to arise from the oxidation of some [Cr_2_As_2_]^2–^ layers containing Cr^2+^ to [Cr_2_OAs_2_]^2–^ layers containing Cr^3+^. This led us to target the title phase Sr_2_CrO_2_Cr_2_OAs_2_, which is the fully oxidized analogue of Sr_2_CrO_2_Cr_2_As_2_ where all [Cr_2_As_2_]^2–^ layers have been oxidized, accompanied by a relative shift of  in the *ab* plane of neighboring [Sr_2_CrO_2_]^2+^ blocks as shown in Figure 1. The structure of this target phase, shown in Figure 1 (right), is analogous to that reported for the cation-defective Ca_2_Fe_2.6_S_2_O_3_,^18^ and the [Cr_2_OAs_2_]^2–^ blocks are the exact anti-type of the [Sr_2_CrO_2_]^2+^ (i.e., [O_2_CrSr_2_]^2+^) blocks. Similar blocks *M*_2_O*Q*_2_ (*M* = transition metal, *Q* = chalcogen or pnictogen) are known.^19,20^ Sr_2_CrO_3_CrAs with the Sr_2_GaO_2_CuS structure was also discovered as a minor phase in the original Sr_2_CrO_2_Cr_2_As_2_ sample,^14^ and here, we report the crystal structure and magnetism of this material in the bulk form. Thicker oxide slabs are present in this compound compared to Sr_2_CrO_2_Cr_2_As_2_, and the Cr^3+^ ions in the oxide layer are now in a square-pyramidal coordination of oxide anions rather than distended CrO_4_As_2_ octahedra.

## Experimental Section

### Synthesis

One gram samples of Sr_2_CrO_2_Cr_2_OAs_2_ and Sr_2_CrO_3_CrAs were synthesized from SrO, Cr_2_O_3_ (Alfa Aesar, 99.6%), Cr (Alfa Aesar, 99.95%), and As (Alfa Aesar, 99%) in the ratios of 6:0.85:7.3:6 and 6:1:4:3, respectively. The mixture for Sr_2_CrO_2_Cr_2_OAs_2_ targeted a stoichiometry of Sr_2_Cr_3_O_2.85_As_2_, and the 5% oxygen deficiency was employed as it increased the phase purity of the sample as proposed by Jiang et al.^21^ in their synthesis of Sr_2_CrO_2_Cr_2_As_2_. SrO had been previously prepared via thermal decomposition of SrCO_3_ (Alfa Aesar, 99.994%) by heating it at 830 °C for 16 h and then at 1100 °C for 4 h, all under dynamic vacuum. Cr_2_O_3_ was also pre-dried in a furnace before use. The reagents were thoroughly ground together using an agate pestle and mortar until the mixtures appeared homogeneous. The powders were then pressed into pellets and sealed inside evacuated silica tubes. The Sr_2_CrO_2_Cr_2_OAs_2_ mixture was heated at 800 °C for 2 h (1 °C min^–1^ ramping rate) then at 1200 °C for 8 h (10 °C min^–1^ ramping rate) and then at 1200 °C for 2 h (10 °C min^–1^ ramping rate), with grinding and re-pelletizing between the two 1200 °C heating steps and quenching in ice water from both 1200 °C heating steps. The 800 °C heating step with slow ramping rate was used to ensure that the As reacted before reaching a high vapor pressure. The Sr_2_CrO_3_CrAs target was quenched in ice water after having been heated at 1200 °C for 24 h (10 °C min^–1^ ramping rate).

### Diffraction

An in-house Bruker D8 Advance Eco diffractometer (using Cu K_α_ radiation) was used to gather X-ray powder diffraction (XRPD) data in order to follow the reactions between heating steps. Data for detailed structural analysis were collected on beamline I11^22^ at the Diamond Light Source using 30 min scans with 0.82 Å X-rays (calibrated precisely using a Si standard at the start of each beam time session) with the high-resolution multi-analyzer crystal (MAC) detector. A position-sensitive detector (PSD) was also used on beamline I11 to gather full diffraction patterns at 190 temperatures while cooling from 600 to 300 K in approximately 1 h. Neutron powder diffraction (NPD) was carried out on the WISH instrument^23^ at the ISIS Facility, where approximately 0.8 g of each material was loaded into vanadium cans, and data were obtained at various temperatures between 7 and 543 K using a cryofurnace to cool down and warm up the samples. The XRPD and NPD data were analyzed by Rietveld refinement using the TOPAS Academic V5 software.^24^

### Transmission Electron Microscopy

Electron diffraction (ED) patterns at room temperature and 100 K were acquired on a Philips CM20 transmission electron microscope operated at 200 kV. High-angle annular dark field (HAADF) and annular bright field (ABF) scanning transmission electron microscopy (STEM) images were acquired at room temperature using a FEI Titan 80-300 “cubed” microscope operated at 300 kV. Specimens for the TEM study were prepared by grinding the material under ethanol and depositing a few drops of the suspension onto a copper grid covered by a holey carbon layer. The specimens were prepared in air.

### Magnetometry

A Quantum Design MPMS-3 SQUID magnetometer was employed to gather magnetometry data for the Sr_2_CrO_2_Cr_2_OAs_2_ sample, and a Quantum Design MPMS-XL SQUID magnetometer was used for the Sr_2_CrO_3_CrAs sample. For measurements below 300 K, around 30 mg (accurately weighed) of sample was loaded into a gelatin capsule. This capsule was then secured in a plastic straw, and the straw was placed inside the instrument. Zero-field-cooled (ZFC) and field-cooled (FC) measurements were carried out in a field of 100 Oe. To take account of minuscule amounts of ferromagnetic impurities in the sample, data were also gathered as a function of temperature at 3 and 4 T in the region where the magnetization varied linearly with field, and the susceptibility of the sample was determined by subtraction. For measurements above 300 K, around 30 mg of sample was pressed in a pellet die to form a bar of material. Alumina cement was used to attach the sample to a heater stick on the MPMS-3 magnetometer, and copper foil was wrapped around the material to reduce radiative heat loss. Measurement of magnetization as a function of temperature (with a field of 100 Oe applied) gave the equivalent of a ZFC curve as the sample was warmed from 300 to 800 K, and a FC curve as the sample was then cooled back down from 800 to 300 K.

### High-Temperature Resistance

Sintered Sr_2_CrO_2_Cr_2_OAs_2_ pellets were electrically characterized using the four-point probe technique as follows. Four aluminum contacts with thickness of 500 nm, length of 4 mm, and width of 1.5 mm were thermally evaporated on the sample surface spaced by 1.33 mm each. A current bias from −1 mA to +1 mA was applied across the outer two contacts, while the voltage was measured across the inner two contacts. Signal generation and measurement was carried out using a Keithley 2401 Source measuring unit controlled via a virtual instrument programmed in LabVIEW. Each current–voltage characteristic was acquired with 60 data points, and the mean resistance calculated from 5 current–voltage measurements. Current–voltage measurements were taken while the sample rested on an aluminum stage where the temperature was controlled via a PID controller from 300 to 770 K using a thermocouple in direct contact with the stage, next to the specimen. Measurements were acquired in temperature intervals of 20 K, and the thermocouple reading was used to ensure the temperature of the stage was stable before data acquisition.

## Results and Discussion

### Compositions and Crystal Structures

Sr_2_CrO_2_Cr_2_OAs_2_, a target identified from the nature of the stacking faults in some regions of Sr_2_CrO_2_Cr_2_As_2_ samples,^14^ was difficult to obtain with high purity. The purest sample reported here contains CrAs, Sr_2_CrO_3_CrAs, and As side phases, which proved difficult to avoid. Numerous attempts using a range of starting materials and heating profiles were attempted. Ultimately, the target phase was made with a fairly high purity (87% of the total mass) by heating to a very high temperature of 1200 °C (the limit for a single-walled silica ampoule), including a 5% oxygen deficiency in the reagent stoichiometry, and quenching in ice water. Even though an oxygen deficiency was used in the starting mixture, there was no evidence in the refinements of the room-temperature XRPD (Figure 4) and NPD (Figure S1) data that the final phase was oxygen-deficient. It is plausible that additional O arises due to reaction with the silica tubes; however, the impurities present are also consistent with a stoichiometric target phase given the slight off-stoichiometry of the reaction mixture. Sr_2_CrO_2_Cr_2_OAs_2_ crystallizes in the *P*4/*mmm* space group and is isostructural with the Ca_2_Fe_3-δ_O_3_(S_1-x_Se_x_)_2_ phases first reported by Zhang et al.^18^ (structure shown in Figure 6). The alternating [Sr_2_CrO_2_]^2+^ and [Cr_2_OAs_2_]^2–^ slabs host distorted *trans*-CrO_4_As_2_ and *trans*-CrO_2_As_4_ octahedra, respectively. The CrO_4_As_2_ octahedra are distended along the Cr–As bonds and are oxide-vertex sharing, whereas the CrO_2_As_4_ octahedra are compressed along the Cr–O bonds and share As_2_O faces.

**Figure 2:**
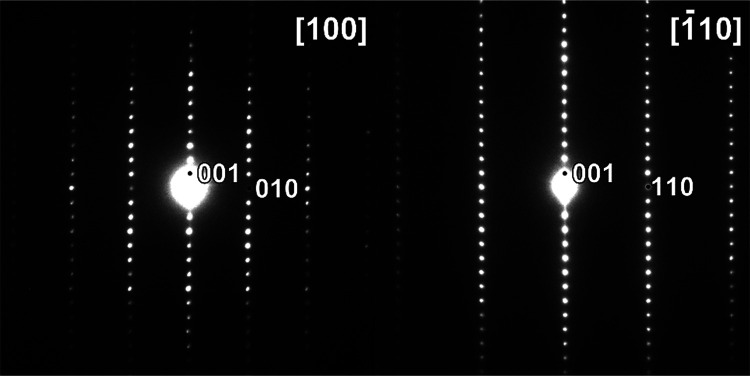
ED patterns taken from [100] and [110] zones of Sr_2_CrO_3_CrAs identified in a bulk sample of Sr_2_CrO_2_Cr_2_As_2_. The measurements were made at 100 K.

**Figure 3:**
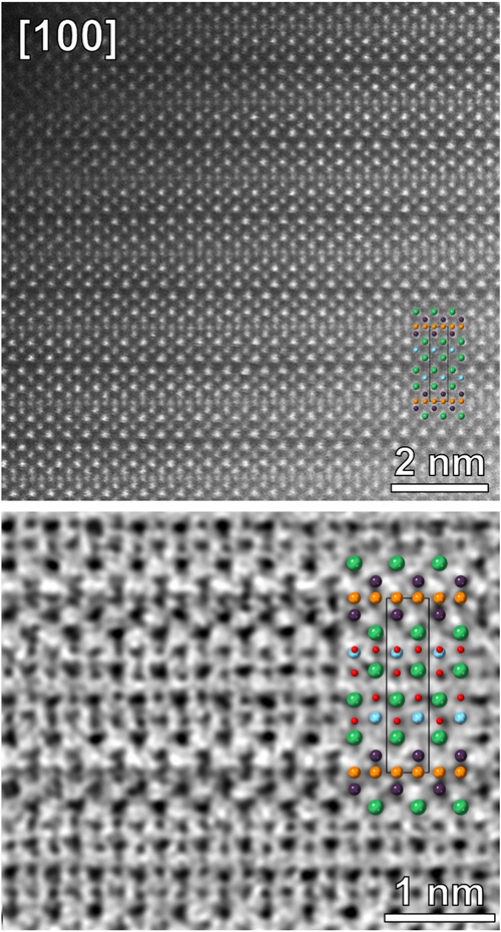
(Top) HAADF-STEM image of a well-ordered Sr_2_CrO_3_CrAs structure identified in a bulk sample of Sr_2_CrO_2_Cr_2_As_2_; (bottom) enlarged fragment of the ABF-STEM image with a Sr_2_CrO_3_CrAs structure overlay. Atoms are Cr1: pale blue; Cr2: dark blue; Sr: green; As: orange; O: red. Unit cell is outlined with a black rectangle.

**Figure 4:**
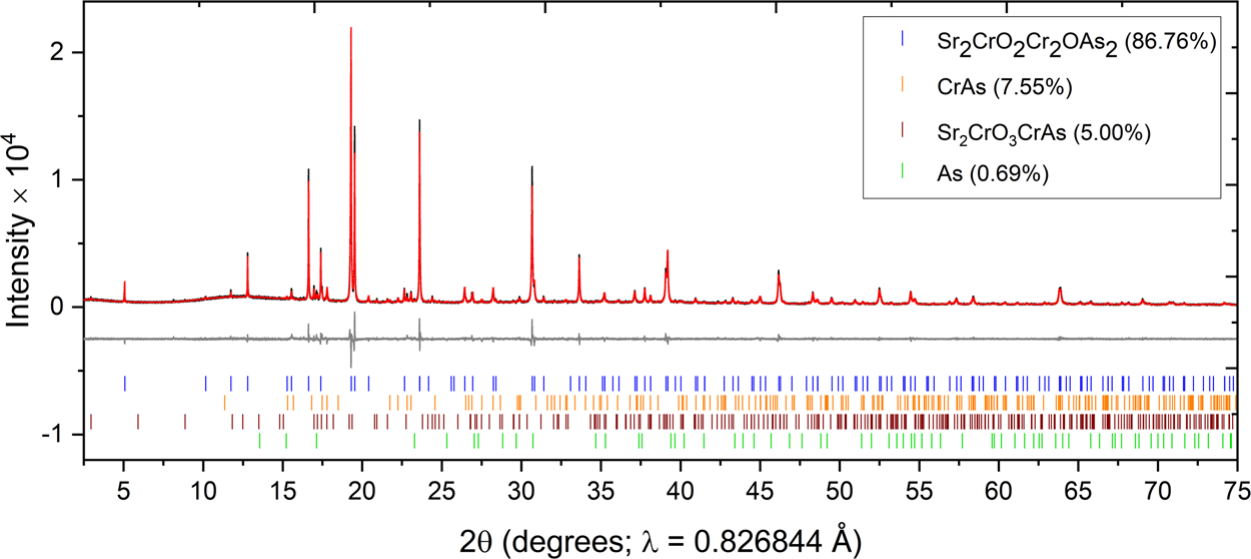
XRPD pattern of Sr_2_CrO_2_Cr_2_OAs_2_ measured at 300 K on the MAC detector at I11 showing the observed (black), calculated (red), and difference (gray) curves. *R*_wp_: 7.679%.

**Figure 5:**
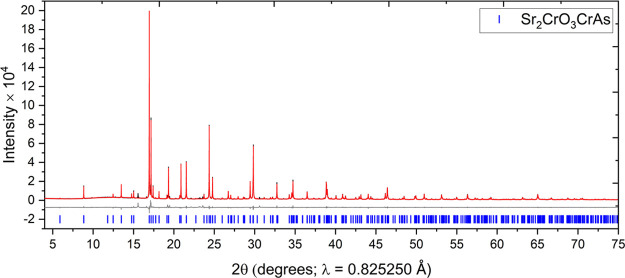
XRPD pattern of Sr_2_CrO_3_CrAs measured at 300 K on the MAC detector at I11 showing the observed (black), calculated (red), and difference (gray) curves. *R*_wp_: 7.915%.

Sr_2_CrO_3_CrAs, which was identified as a minority phase in samples of Sr_2_CrO_2_Cr_2_As_2_ by electron microscopy (Figures 2 and 3), was synthesized successfully with only very minor amounts of unidentifiable side phases, as shown by the XRPD (Figures 4 and 5) and NPD (Figure S2) data at 300 K. It adopts the Sr_2_GaO_3_CuS structure type (Figure 7) with the *P*4/*nmm* space group. Cr^2+^ exists in the anti-PbO-type [CrAs]^−^ layers (tetrahedrally coordinated by As), whereas Cr^3+^ is present in the [Sr_2_CrO_3_]^+^ blocks (in a CrO_5_ square-pyramidal coordination). Low-temperature electron diffraction measurements of this phase did not reveal any structural changes down to 100 K (Figure 2).

### Structure Refinement

**Figure 6:**
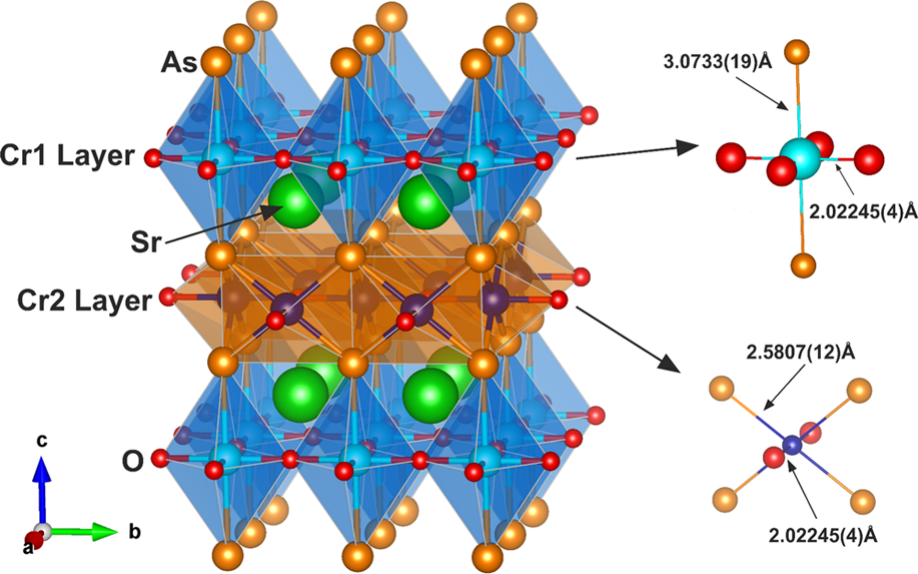
Structure of Sr_2_CrO_2_Cr_2_OAs_2_. The CrO_4_As_2_ and CrO_2_As_4_ distorted octahedra are shown by the blue and orange polyhedra, respectively. Ellipsoids with 99% displacement (right) are given using isotropic displacement parameters refined from room-temperature WISH NPD data (detailed in Table S1). Atoms are Cr1: pale blue; Cr2: dark blue; Sr: green; As: orange; O: red.

**Figure 7:**
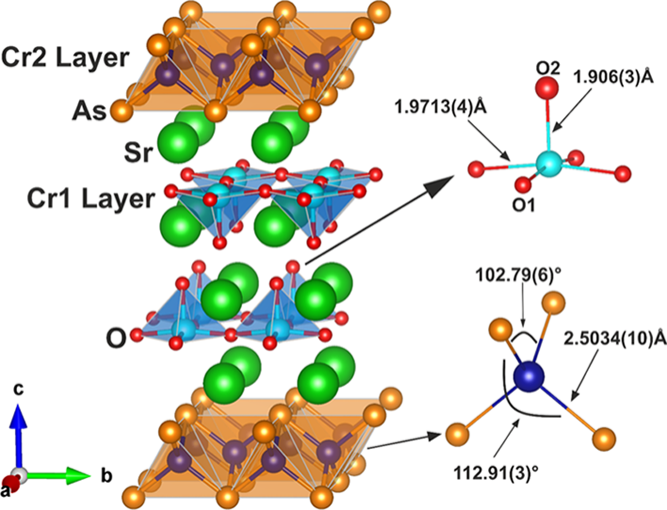
Structure of Sr_2_CrO_3_CrAs. The CrO_5_ square-pyramids and CrAs_4_ tetrahedra are shown by the blue and orange polyhedra respectively. Ellipsoids with 99% displacement (right) are given using isotropic displacement parameters refined from room-temperature WISH NPD data (detailed in Table S2). Atoms are Cr1: pale blue; Cr2: dark blue; Sr: green; As: orange; O: red.

The values in Table 1 and Figures 6 and 7 (refined from synchrotron XRPD data and WISH NPD data, respectively) detail the lattice parameters, atomic positions, and a selection of bond lengths and angles corresponding to both the Sr_2_CrO_2_Cr_2_OAs_2_ and Sr_2_CrO_3_CrAs phases. A comparison between values refined from XRPD and NPD measurements are given in the Supporting Information (Tables S1 and S2). Each site occupancy factor was allowed to freely refine in the early stages of the refinement, but there was no significant deviation from the ideal value of 1 by any atom, so these values were then fixed.

**Table 1:** Refinement Results from XRPD Patterns Collected at 300 K Using the MAC Detector at I11

	Sr_2_CrO_2_Cr_2_OAs_2_	Sr_2_CrO_3_CrAs
diffractometer	I11 (MAC)	I11 (MAC)
wavelength (Å)	0.826844	0.825250
temperature (K)	300	300
space group	*P*4/*mmm*	*P*4/*nmm*
*a* (Å)	4.040319(16)	3.909877(13)
c (Å)	9.33140(7)	16.05417(7)
*V* (Å^3^)	152.327(2)	245.422(2)

Sr_2_CrO_2_Cr_2_OAs_2_								
atom	site	*x*	*y*	*z*	occupancy	*U*_11_ (Å^2^)	*U*_22_ (Å^2^)	*U*_33_ (Å^2^)
Sr	2*h*	0.5	0.5	0.17664(8)^b^	1	0.0024(2)	=*U*_11_	0.0114(6)
Cr1	1*a*	0	0	0	1	0.0000(4)	=*U*_11_	0.0171(9)
Cr2	2*e*	0.5	0	0.5	1	0.0002(3)	=*U*_11_	0.0134(6)
O1	2*f*	0.5	0	0	1	0.0063(8)^a^	=*U*_11_	=*U*_11_
O2	1*d*	0.5	0.5	0.5	1	0.0063(8)^a^	=*U*_11_	=*U*_11_
As	2*g*	0	0	0.32192(8)	1	0.0000(3)	=*U*_11_	0.0125(7)

Sr_2_CrO_3_CrAs								
atom	site	*x*	*y*	*z*	occupancy	*U*_11_ (Å^2^)	*U*_22_ (Å^2^)	*U*_33_ (Å^2^)
Sr1	2*c*	0.75	0.75	0.19950(5)	1	0.0053(4)	=*U*_11_	0.0089(6)
Sr2	2*c*	0.75	0.75	0.41633(5)	1	0.0063(4)	=*U*_11_	0.0093(6)
Cr1	2*c*	0.25	0.25	0.31312(8)	1	0.0039(5)	=*U*_11_	0.0074(9)
Cr2	2*a*	0.25	0.75	0	1	0.0081(5)	=*U*_11_	0.0100(8)
O1	4*f*	0.25	0.75	0.29518(19)	1	0.0130(10)	=*U*_11_	=*U*_11_
O2	2*c*	0.25	0.25	0.4295(3)	1	0.0101(13)	=*U*_11_	=*U*_11_
As	2*c*	0.25	0.25	0.09633(5)	1	0.0075(4)	=*U*_11_	0.0077(6)

a These oxygen displacement parameters were fixed at the same value, and they were refined isotropically.

b The estimated standard deviations on the refined parameters produced in a Rietveld refinement give an indication of the data quality and may underestimate the true experimental uncertainty in a refined value.

### Crystal Structures

Sr_2_CrO_3_CrAs is one of a wide variety of compositions that are known to adopt the Sr_2_GaO_3_CuS^25^ structure. These include materials such as Sr_2_MnO_3_CuS,^26^ Sr_2_CrO_3_CuS,^27^ and Ca_2_FeO_3_Cu*Ch* (*Ch* = S, Se).^28^ Furthermore, in terms of the individual alternating blocks, the anti-PbO-type chromium arsenide layers found in this compound are well documented in the literature. For example, Park et al.^29^ report on the nuclear and magnetic structures of LaCrAsO, containing alternating [LaO]^+^ and [CrAs]^−^ blocks where the chromium ions are in the +2 oxidation state. The Cr–As length refined from XRPD is given as 2.494(1) Å, and this is comparable to the value of 2.4927(5) Å found here for Sr_2_CrO_3_CrAs (Table S2). This suggests that these chromium arsenide layers in Sr_2_CrO_3_CrAs host the same Cr^2+^ species and that the oxide layer sitting between the [CrAs]^−^ layers has little effect on the nature of the Cr–As bonding. The CrO_5_ square-pyramidal environment in the [Sr_2_CrO_3_]^+^ block of Sr_2_CrO_3_CrAs is less common, but it is known in Sr_2_CrO_3_CuSe^17^ where the axial Cr-O bond (aligned along the *c* axis) and basal Cr–O bonds (aligned roughly within the *ab* plane) have lengths of 1.999(15) and 1.9830(14) Å, respectively. There is significant deviation from this in the Sr_2_CrO_3_CrAs compound, where the corresponding Cr–O distances are 1.868(5) and 1.9760(5) Å. This shows that the chromium oxide layer is not particularly rigid as the CrO_5_ square-pyramids are susceptible to significant changes in shape. However, the bond valence sums (calculated using the Cr–O bond lengths refined from XRPD data and using reference bond length data provided by Brown and Altermatt^30^) predict chromium oxidation states of +2.46(6) (Sr_2_CrO_3_CuSe) and +2.70(2) (Sr_2_CrO_3_CrAs); therefore, it is highly probable that these two systems contain similar chromium species in the CrO_5_ environments.

Sr_2_CrO_2_Cr_2_OAs_2_ adopts the lesser-explored structure type formally derived by oxygen insertion (Figure 1) from that of Sr_2_CrO_2_Cr_2_As_2_, the well-known structure of which was first reported for *A*_2_MnO_2_Mn_2_*B*_2_ (*A* = Sr, Ba; *B* = As, Sb, Bi) by Brechtel et al.^31^ Comparisons can be made by again focusing on each type of layer in the structure. We previously reported that the Cr–O distance within the CrO_2_ square-planar sheets of Sr_2_CrO_2_Cr_2_As_2_ refined to a value of 2.00400(1) Å^14^ (equal to half the basal lattice parameter), and in the case of Sr_2_CrO_2_Cr_2_OAs_2_, we find a similar value of 2.0202(1) Å for this Cr1–O distance. The formal oxidation of the Cr_2_As_2_ layer to Cr_2_OAs_2_ therefore seems to leave these CrO_2_ sheets unaffected, as would be expected due to the largely unchanged Cr1 environment. Sr_2_CrO_2_Cr_2_OAs_2_ is the first reported example of Cr_2_OAs_2_ layers; however, some iron oxychalcogenides have been discovered with analogous Fe_2_O*Ch*_2_ (*Ch* = S, Se) environments, such as *A*_2_F_2_Fe_2_O*Q*_2_ (*A* = Sr, Ba; *Q* = S, Se)^32^ and La_2_O_2_Fe_2_O*Ch*_2_ (*Ch* = S, Se).^33−35^ Further examples where layers of this type host alternative 3*d* transition metals include the Mn_2_OSe_2_ environments in *A*_2_O_2_Mn_2_OSe_2_ (*A* = La, Ce, Pr),^19,36^ the Co_2_OSe_2_ environments in La_2_O_2_Co_2_OSe_2_,^19,37^ and the Ti_2_OAs_2_ environments in Ba_2_Ti_2_OAs_2_Cr_2_As_2_,^11^ BaTi_2_OAs_2_,^12^ and Ba_2_Ti_2_OAs_2_Fe_2_As_2_.^20^

### Magnetic Ordering in Sr_2_CrO_2_Cr_2_OAs_2_

**Figure 8:**
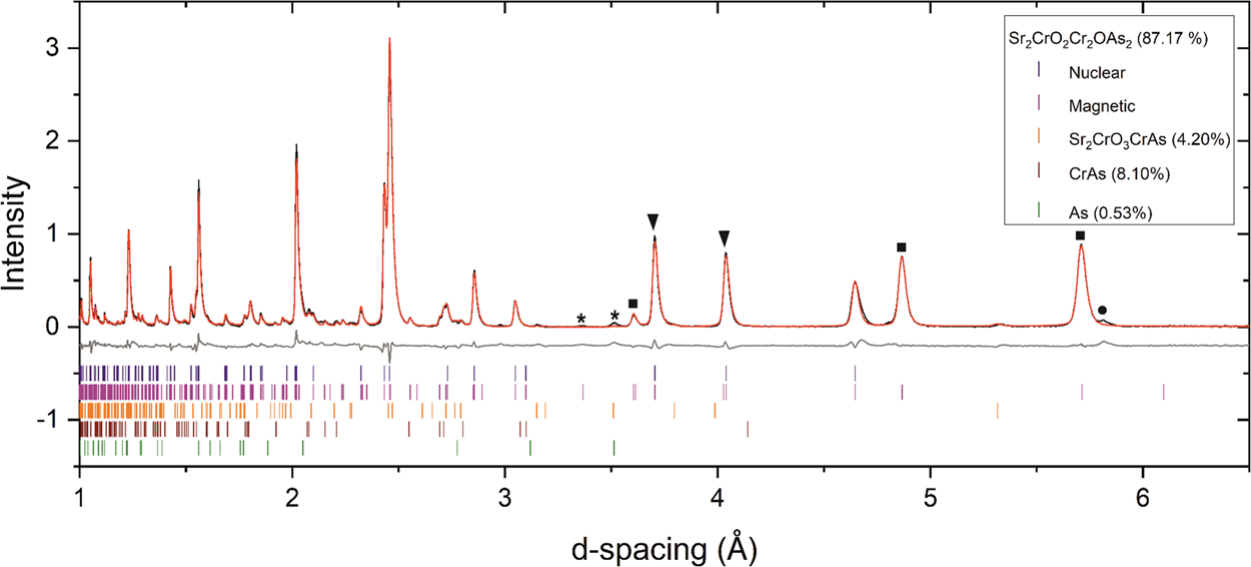
NPD pattern of Sr_2_CrO_2_Cr_2_OAs_2_ (combination of banks 3 and 8 with average 2θ = 90°) measured at 10 K on the WISH instrument at ISIS showing the observed (black), calculated (red), and difference (gray) curves. The black triangles and black squares denote reflections with *k* = (0 0 0) and , respectively. The asterisks give examples of peaks that disappear before 300 K and are due to magnetic order in CrAs. The black circle highlights an unidentified impurity peak, which is presumably nuclear (not magnetic) in origin as it does not change intensity with varying temperature. *R*_wp_: 5.751%.

**Figure 9:**
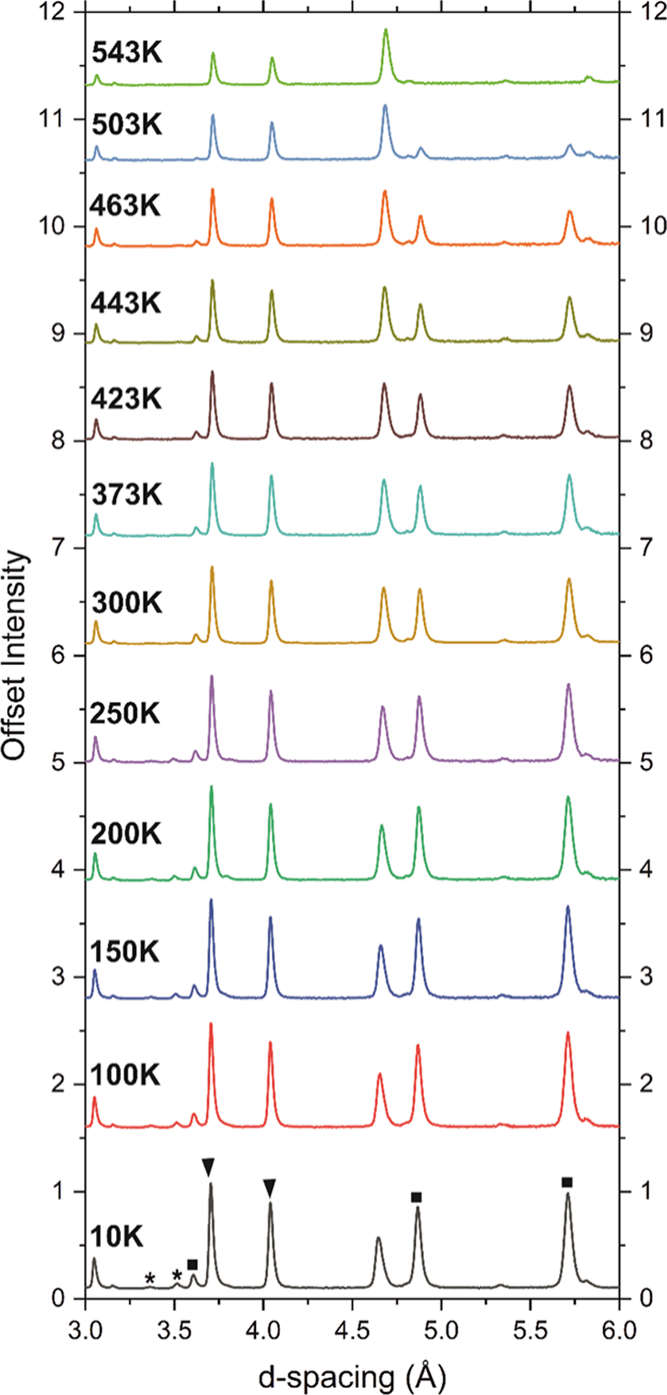
NPD patterns of Sr_2_CrO_2_Cr_2_OAs_2_ (combination of banks 3 and 8 with average 2θ = 90°) at different temperatures measured on the WISH instrument at ISIS showing the evolution of the magnetic peaks. Magnetic Bragg peaks labeled with black squares disappear between 503 and 543 K, whereas those labeled by black triangles are still present at 543 K. Those highlighted by asterisks can be explained as the magnetic Bragg peaks of CrAs (*T*_N_ ≈ 300 K).

NPD data collected on the Sr_2_CrO_2_Cr_2_OAs_2_ sample at 10 K (Figure 8 and Figure S8) show a series of reflections where the intensities and *d*-spacings cannot be accounted for from scattering due to the nuclear model alone. These additional peaks only occur at long *d*-spacings, which is indicative of a magnetic origin. These peaks decrease in intensity as the sample is warmed (Figure 9) and can be explained by scattering from arrays of long-range ordered magnetic moments. The reflections labeled by black triangles in Figure 8 are positioned on top of nuclear peaks and are therefore accounted for by the *k* = (0 0 0) propagation vector. In contrast, the *k*-vector of the peaks denoted by a black square is , and these imply that the cell of the magnetic structure is a 2*a*_nuc_ × 2*a*_nuc_ × *c*_nuc_ expansion of the nuclear unit cell and that the two Cr sublattices order independently with different propagation vectors. A number of much less intense magnetic Bragg reflections are highlighted by asterisks in Figure 8 and disappear on warming between 200 and 300 K. These can be attributed to the magnetic structure of the CrAs side phase evident in the XRPD pattern, which has a Néel temperature of approximately 300 K. The magnetic ordering transitions of Sr_2_CrO_2_Cr_2_OAs_2_ are not detected in the high-temperature magnetometry data (Figures S3 and S4).

**Figure 10:**
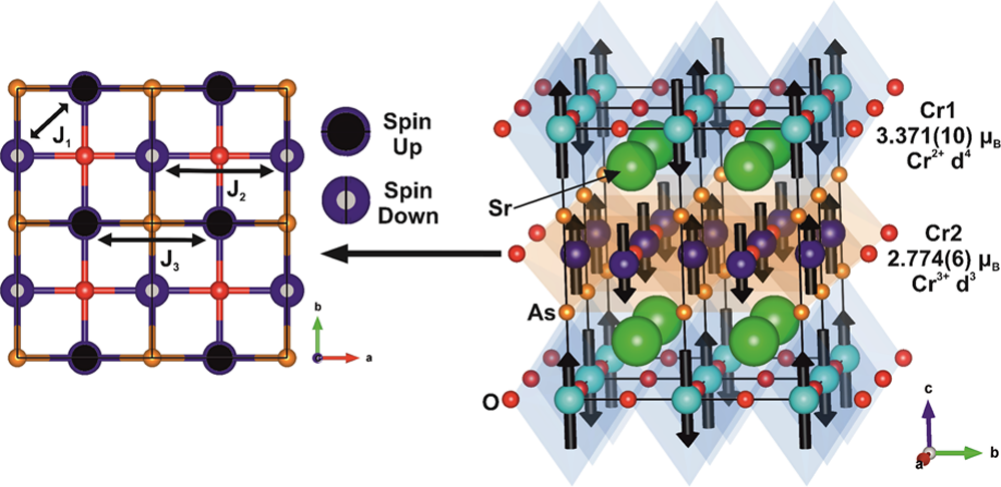
Model for the magnetic order on the Cr1 oxide-rich sublattice and Cr2 arsenide-rich sublattice of Sr_2_CrO_2_Cr_2_OAs_2_ at 10 K. The major magnetic interactions in the Cr2 layer as considered for materials with analogous layers (examples including Ni et al.^36^ Kabbour et al.^32^ Free et al.^19^ and Wang et al.^40^) are labeled J_1_ (Cr–Cr direct exchange), *J*_2_ (180° Cr–O–Cr superexchange) and *J*_3_ (∼100° Cr–As–Cr superexchange).

The ISODISTORT package^38^ was used to deduce the magnetic modes available to this system, and then Rietveld refinement was carried out to assess the suitability of each mode to fit the NPD data. These modes are symmetry-adapted linear combinations of the Cr magnetic moments, which enable refinement of the magnetic structure with symmetry imposed. The most suitable model was one that describes the Cr1 layer moments using a single mode (mM3 + A2(a,0)) and the Cr2 layer moments using a single mode (mΓ4 + B1(a,0)) (these modes are depicted in Figure 10). The resulting model has all the Cr moments aligned along the *c* axis in antiferromagnetic arrangements, which is consistent with the absence of any intensity on reflections indexed perpendicular to the *c* axis (i.e., (00l) reflections). Nearest neighbors in the Cr1 sublattice (CrO_2_ layers) are coupled antiferromagnetically, as is expected for σ- and π-type superexchange interactions between high spin *d*^4^-*d*^4^ ions in co-aligned distended octahedral coordination mediated by the 2*p* orbitals of the O^2–^ anion. Reference (14) discusses the degree of distention of the CrO_4_As_2_ octahedra in relation to the expected Jahn–Teller distortion for a Cr^2+^*d*^4^ ion.

The model for the Cr2 sublattice (Cr_2_OAs_2_ layers) in Sr_2_CrO_2_Cr_2_OAs_2_ also has antiferromagnetic coupling of nearest neighbor Cr^3+^ moments. The main competing magnetic interactions are highlighted by Figure 10 (as previously described by Ni et al.^36^): the direct exchange between nearest neighbor Cr^3+^ centers (*J*_1_), the 180° superexchange mediated by O^2–^ (*J*_2_), and the approximately 100° superexchange mediated by As^3–^ (*J*_3_). It seems that this system is dominated by the direct exchange interaction (*J*_1_) between the *d_xz_* and *d_yz_* orbitals of neighboring Cr centers, which is antiferromagnetic in nature. This interaction acts between all the Cr^3+^ ions that are nearest neighbors, forming a checkerboard pattern of Cr^3+^ spins aligned along the *c* axis and alternating in their relative directions. The model suggests that this direct exchange is the strongest coupling mechanism, exceeding the strength of the superexchange interactions. The linear superexchange (*J*_2_) involving empty *d*_*z*^2^_ orbitals is predicted to be an antiferromagnetic interaction by application of the Goodenough–Kanamori rules; however, here, the moments are ferromagnetically aligned along that pathway. The other superexchange interaction (*J*_3_) involving empty *d_xy_* orbitals is predicted to be antiferromagnetic in nature, but again, here, moments connected by this interaction are aligned to be ferromagnetic. Stock and McCabe^7^ summarize a number of long-range magnetic ordering schemes reported for layered materials comprising *M*_2_OSe_2_ (*M* = transition metal) blocks. One observation of interest is that the magnetic structure for the Cr2 sublattice in the Cr_2_OAs_2_ layers of Sr_2_CrO_2_Cr_2_OAs_2_ is similar to that described for La_2_O_2_Mn_2_OSe_2_,^19,36^ containing Mn_2_OSe_2_ layers similar to the Cr_2_OAs_2_ layers considered here, where the *d*^5^ moments for Mn^2+^ ions are also directed perpendicular to the layers and order in a similar checkerboard manner. The magnetic structure of La_2_O_2_Mn_2_OSe_2_ was also proposed to be a consequence of the antiferromagnetic nearest neighbor direct exchange interactions being dominant and frustrating the *J*_2_ and *J*_3_ superexchange interactions, resulting in ferromagnetic alignment of the moments along the perpendicular -Mn-O-Mn-O-Mn- chains.

The significantly larger refined long-range ordered moment per Cr ion in the Cr1 layer of 3.371(10) μ_B_ compared to the 2.774(6) μ_B_ per Cr in the Cr2 layer are consistent with the assignment of these as Cr^2+^*d*^4^ and Cr^3+^*d*^3^ cations respectively, with the ordered moments reduced below the maximum expected spin-only values of 4 and 3 μ_B_ respectively by covalency. The Cr1 (Cr^2+^) moment is similar in direction and magnitude to the Cr^2+^ moment in the analogous layers in both Ba_2_CrO_2_Cr_2_As_2_ and Sr_2_CrO_2_Cr_2_As_2_,^14,15^ where the CrO_2_ layers are also antiferromagnetic, therefore also supporting the assignment of the Cr1 oxidation state as Cr^2+^. Furthermore, bond valence sums (calculated using bond length data provided by Brese and O’Keefe^39^ and where the literature Cr–As bond length used was that for Cr^II^–As in both cases as a known Cr^III^–As bond length was not found) corroborate with this assignment as these give Cr1 an oxidation state of +2.090(2) and Cr2 an oxidation state of +2.980(8). The observed antiferromagnetism, the bond valence sums, and the sizes of the ordered moments are consistent with a lack of mixed valency on the Cr1 and Cr2 sites.

**Figure 11:**
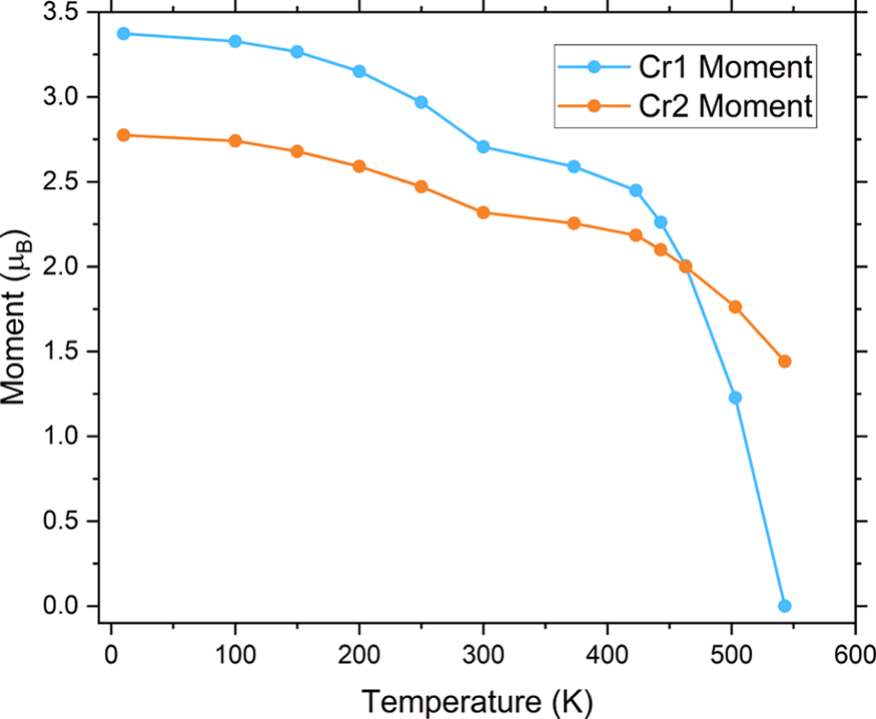
Value of the Cr1 and Cr2 moments (per Cr ion) in Sr_2_CrO_2_Cr_2_OAs_2_ refined from NPD data collected at different temperatures on the WISH instrument at ISIS. The ESD obtained from the refinement on each value of the moment is smaller than the width of the corresponding data point. The apparent dip in the value of the moments at 300 K is due to the change in sample environment and poor temperature calibration close to room temperature for the high temperature sample environment.

Figure 11 displays the refined value of the Cr moment in each layer in Sr_2_CrO_2_Cr_2_OAs_2_ as the temperature is increased. Upon warming the sample, it is the Cr1 (Cr^2+^) moments that lose long-range order first and have the lower *T*_N_ of 530(10) K, with the *T*_N_ of the Cr2 (Cr^3+^) moments predicted to be approximately 600 K based on the evolution of the moment with temperature. The long-range ordering of the Cr^3+^ Cr2 moments in the Cr_2_OAs_2_ layers occurs at a similar temperature to the Cr^2+^ moments in the [Cr_2_As_2_]^2–^ layers of Sr_2_CrO_2_Cr_2_As_2_^14^ and with a similar magnitude of the long-range ordered moment. This is consistent with a stronger reduction of the ordered Cr^2+^ moment in the [Cr_2_As_2_]^2–^ layers in Sr_2_CrO_2_Cr_2_As_2_ due to covalency, with evidence for some delocalization of electrons in that compound suggested by the fact that the compound is metallic.^21^

**Figure 12:**
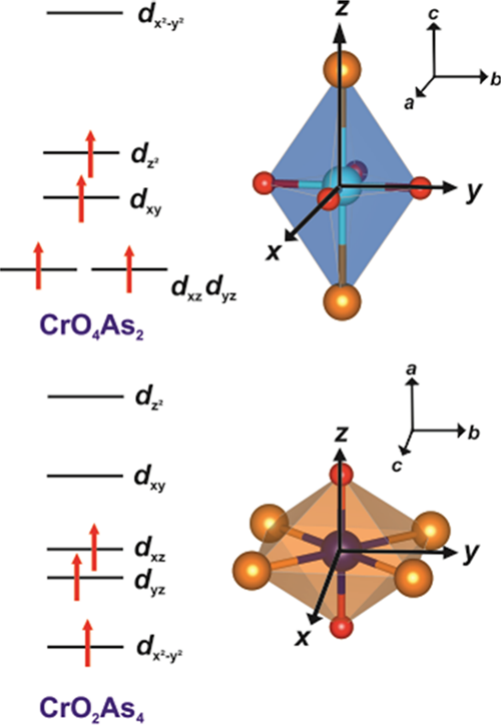
Ligand field splitting schemes for the CrO_4_As_2_ and CrO_2_As_4_ distorted octahedra. The *x*, *y*, and *z* axes shown, which apply to these local coordinations, correspond to the *a*, *b*, and *c* crystallographic axes respectively for the CrO_4_As_2_ case and the *c*, *b*, and *a* crystallographic axes respectively for the CrO_2_As_4_ case. The ligand field splitting for the CrO_2_As_4_ case is as described for analogous CoO_2_Se_4_ octahedra in La_2_O_2_Co_2_OSe_2_ by Wu *et al.*(42)

Whangbo et al.^41^ describe a method by which the spin direction in a magnetically ordered system can be related to the ligand field of the magnetic ion (Figure 12) using spin-orbit coupling arguments. In the CrO_4_As_2_ distended octahedra (Cr1), the change in the magnetic quantum number between the HOMO and LUMO is given by |Δ*L_z_*| = 0, predicting that the moments order parallel to the principal axis (along the crystallographic *c*-direction, perpendicular to the layers). As for the CrO_2_As_4_ environments (Cr2), this change in the magnetic quantum number between the HOMO and LUMO is given by |Δ*L_z_*| = 1, therefore predicting that the moments order perpendicular to the principal axis, which is defined in Figure 12 as parallel to the Cr2–O bonds, and thus, the moments are permitted to lie parallel to the crystallographic *c* axis and thus perpendicular to the layers as observed. It is reported that La_2_O_2_Fe_2_OSe_2_^34^ and La_2_O_2_Co_2_OSe_2_^19,37^ have transition metal moments oriented in the *ab* plane, whereas La_2_O_2_Mn_2_OSe_2_^19,36^ contains moments directed along the *c* axis. The FeO_2_Se_4_ and CoO_2_Se_4_ coordination environments have |Δ*L_z_*| = 1 and |Δ*L_z_*| = 0, respectively. Therefore, the Fe compound is predicted to contain moments perpendicular to *z* and the Co compound moments parallel to *z* (where *z* is parallel to the O-*M*-O direction of the *M*O_2_Se_4_ octahedron as defined in Figure 12). This agrees with the models determined by experiment because moments perpendicular and parallel to *z* can both lie in the crystallographic *ab* plane. The Mn^2+^*d*^5^ ion is predicted to have little spin direction preference. Overall, the transition metal moments in these *M*_2_O*Q*_2_ (*M* = transition metal; *Q* = chalcogen or pnictogen) layers lie perpendicular to the crystallographic *ab* plane for Cr^3+^ (*d*^3^) and Mn^2+^ (*d*^5^) and lie within the *ab* plane for Fe^2+^ (*d*^6^) and Co^2+^ (*d*^7^).

### Magnetic Ordering in Sr_2_CrO_3_CrAs

**Figure 13:**
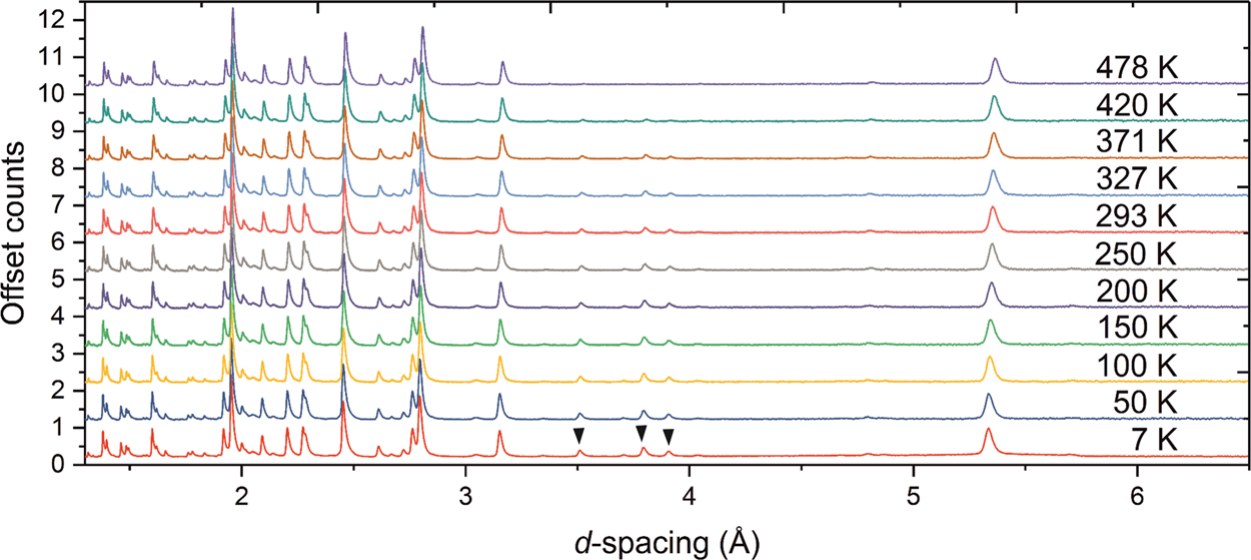
NPD patterns of Sr_2_CrO_3_CrAs (combination of banks 3 and 8 with average 2θ = 90°) at different temperatures measured on the WISH instrument at ISIS showing the evolution of the magnetic peaks. Magnetic Bragg peaks of the main phase are denoted by the black triangles.

**Figure 14:**
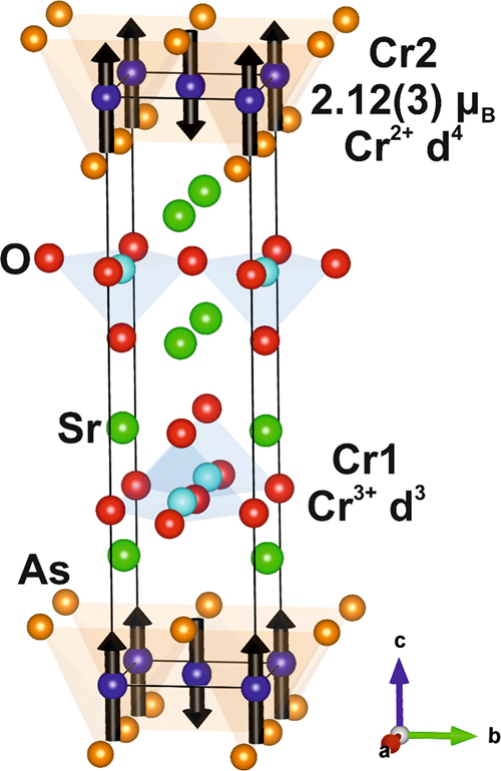
Model for the magnetic order in Sr_2_CrO_3_CrAs at 7 K. The magnetic unit cell shown has the same *a*_nuc_ × *a*_nuc_ × *c*_nuc_ dimensions as the nuclear unit cell.

**Figure 15:**
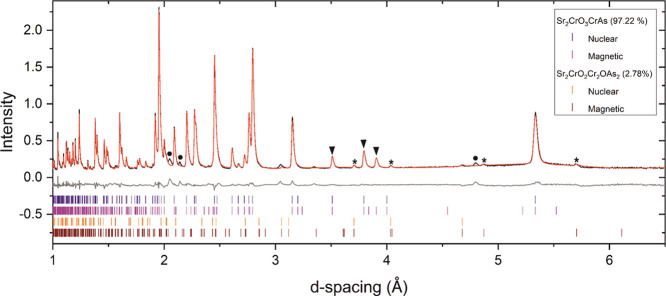
NPD pattern of Sr_2_CrO_3_CrAs (combination of banks 3 and 8 with average 2θ = 90°) measured at 7 K on the WISH instrument at ISIS showing the observed (black), calculated (red), and difference (gray) curves. The reflections labeled with a black triangle correspond to magnetic Bragg peaks arising from antiferromagnetic order in the arsenide layer. The asterisks denote magnetic peaks arising from the Sr_2_CrO_2_Cr_2_OAs_2_ impurity phase. The black circles highlight unidentified impurity peaks, which are presumably nuclear (not magnetic) in origin as their intensities do not change with varying temperature. *R*_wp_: 4.620%.

Variable-temperature NPD studies were also performed for Sr_2_CrO_3_CrAs. Reflections due to long range antiferromagnetic ordering are again observed, and these decrease in intensity until 478 K (Figure 13), at which temperature there are no longer any additional peaks than those arising from the nuclear model. Refinement of the mΓ2- (a,0) mode, with *k* = (0 0 0), gives the best-fitting model, and this consists of antiferromagnetically ordered Cr^2+^ moments in the [CrAs]^−^ layer aligned along the *c*-direction as depicted in Figure 14. In the 7 K refinement (Figure 15 and Figure S9), the long-range-ordered moment per Cr^2+^ ion is 2.12(3) μ_B_, which is lower than the predicted value of 4 μ_B_ for a *d*^4^ ion due to significant covalency in the Cr–As bonds. It is comparable to the saturation value of 2.298(8) μ_B_ per Cr in the similar [Cr_2_As_2_]^2–^ layers of Ba_2_CrO_2_Cr_2_As_2_.^14^

**Figure 16:**
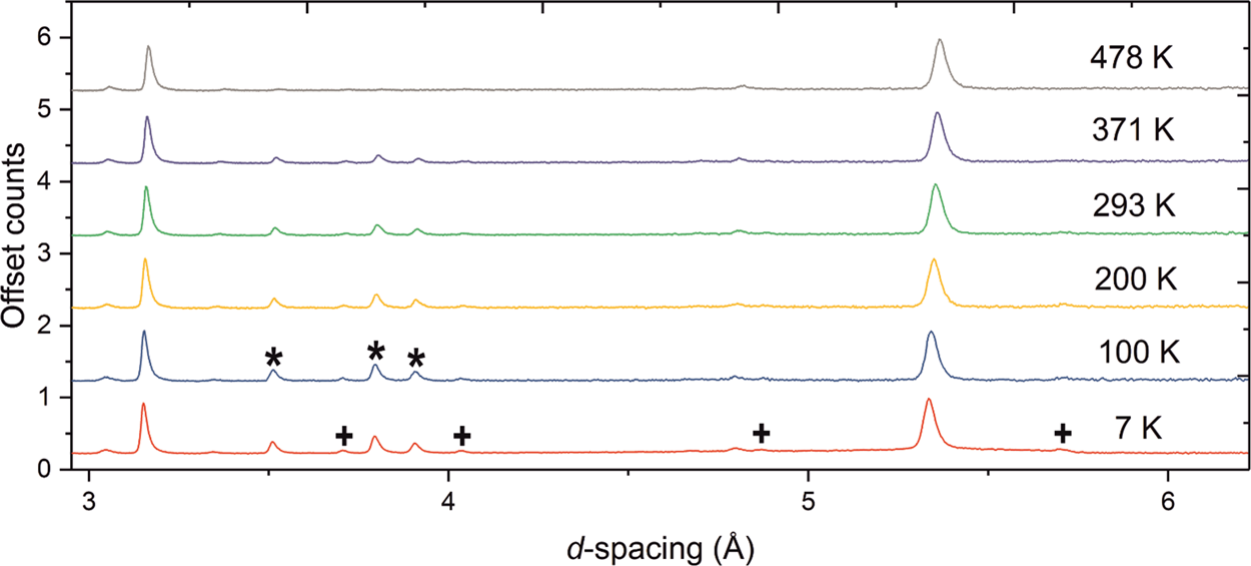
NPD patterns of Sr_2_CrO_3_CrAs (combination of banks 3 and 8 with average 2θ = 90°) at different temperatures measured on the WISH instrument at ISIS showing the magnetic Bragg peaks of the main phase Sr_2_CrO_3_CrAs (*) and the Sr_2_CrO_2_Cr_2_OAs_2_ side phase (+).

In the high *d*-spacing region of the NPD data, some very small peaks can be indexed as the magnetic reflections of a Sr_2_CrO_2_Cr_2_OAs_2_ impurity phase (Figure 16). Although this phase is not detected in the XRPD data, its presence is found due to the presence of these magnetic peaks at high *d*-spacing where reflections are generally fewer in number and more dispersed in *d*-spacing. The nuclear reflections of this impurity phase overlap with those of the main phase and are difficult to observe due to their relatively low intensities.

**Figure 17:**
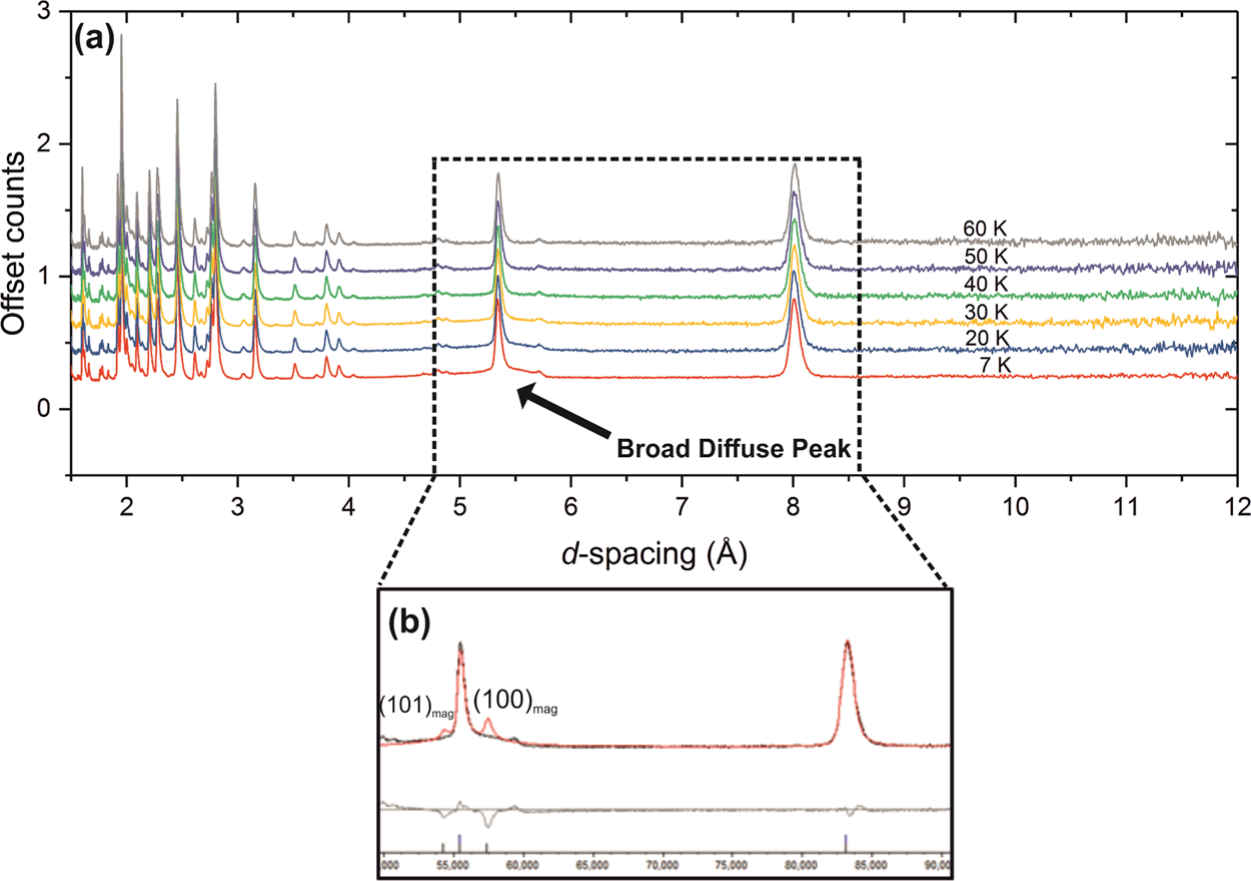
(a) NPD patterns of Sr_2_CrO_3_CrAs (combination of banks 3 and 8 with average 2θ = 90°) at low temperatures measured on the WISH instrument at ISIS and (b) simulated magnetic peaks (red) of Sr_2_CrO_3_CrAs when the oxide layer contains moments aligned in a similar manner to Sr_2_CrO_3_FeAs,^47^ showing that the broad diffuse peak may arise due to short-range order of the Cr^3+^ oxide layer moments in the *ab* plane.

The orientation of the Cr^2+^ moments in the [CrAs]^−^ layer along the *c*-direction is replicated by magnetic ions in a number of related systems containing anti-PbO-type transition metal arsenide layers. Examples include the Mn^2+^ moments in LaMnAsO,^43^ BaMn_2_As_2_,^44^ and Sr_2_MnO_2_Mn_2_As_2_^45^ and the Cr^2+^ moments in LaCrAsO,^29^ BaCr_2_As_2_,^46^ and Ba_2_CrO_2_Cr_2_As_2_.^14^ Other materials adopting the Sr_2_GaO_3_CuS structure type, such as Sr_2_CrO_3_FeAs^47^ and Sr_2_CrO_3_CuSe,^17^ exhibit long-range antiferromagnetic ordering of the nearest-neighbor Cr^3+^ moments in the oxide layer. However, unlike these examples, the Cr^3+^ cations in the oxide layer of Sr_2_CrO_3_CrAs do not contribute to sharp magnetic Bragg peaks, and so the nature of the magnetic ordering must differ here. Instead, diffuse scattering can be seen around 5.33 Å *d*-spacing below 40 K—a position comparable to the main magnetic reflections observed for Sr_2_CrO_3_FeAs^47^ (see Figure 17 and Figure S7). This could explain the broad signal observed in the magnetometry data (Figure S5). It may be the case that there exists some short-range order of the Cr^3+^ moments in the oxide layer, where the moments are antiferromagnetically aligned in the *ab* plane.

### Structural Distortion in Sr_2_CrO_2_Cr_2_OAs_2_

**Figure 18:**
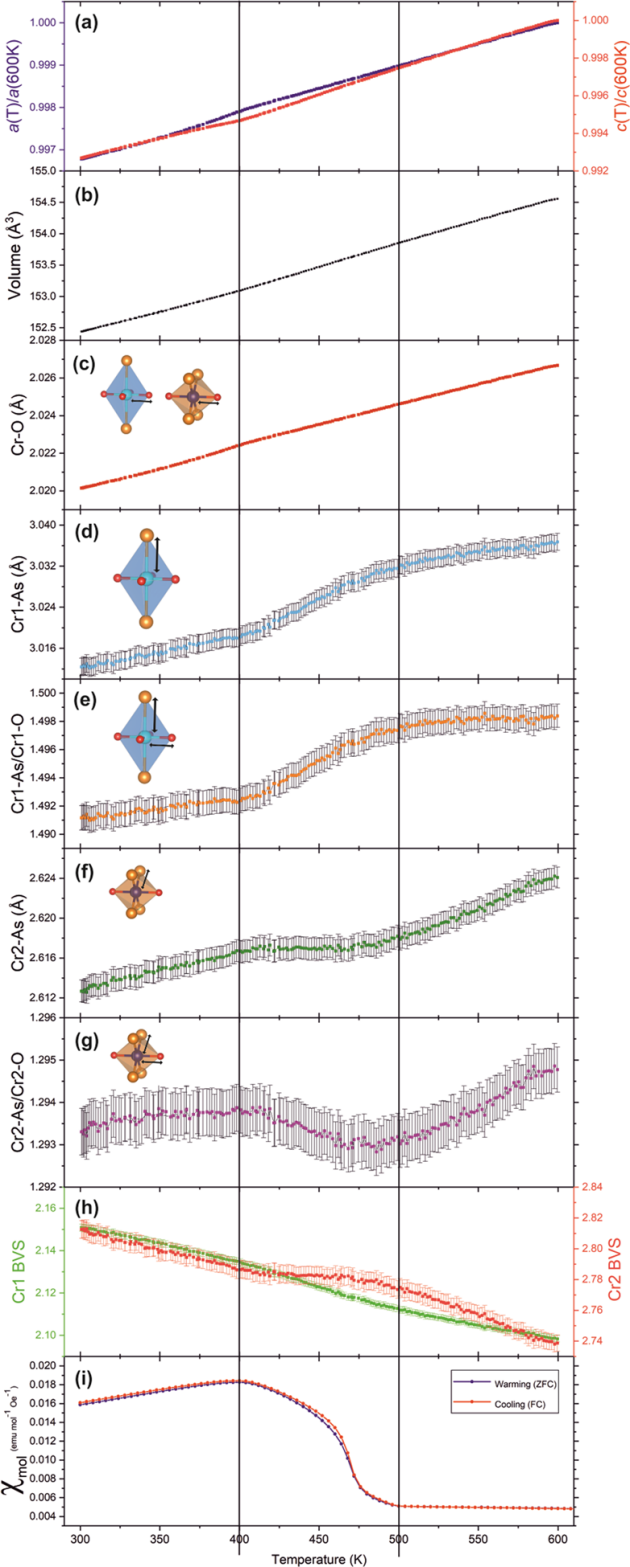
Refinement results of the variable-temperature XRPD data of Sr_2_CrO_2_Cr_2_OAs_2_ showing changes in (a) lattice parameters *a* and *c* normalized against their value at 600 K, (b) unit cell volume, (c) Cr–O distances (identical for Cr1-O and Cr2-O), (d) Cr1-As distance, (e) Cr1-As/Cr1-O bond length ratio, (f) Cr2-As distance, (g) Cr2-As/Cr2-O bond length ratio, and (h) Cr1 and Cr2 bond valence sum (BVS) (calculated using bond length data provided by Brese and O’Keefe^39^ and where the literature Cr–As bond length used was that for Cr^II^–As in both cases as a known Cr^III^–As bond length was not found). High-temperature zero-field-cooled (ZFC) and field-cooled (FC) curves, measured in a field of 100 Oe, are given in (i).

**Figure 19:**
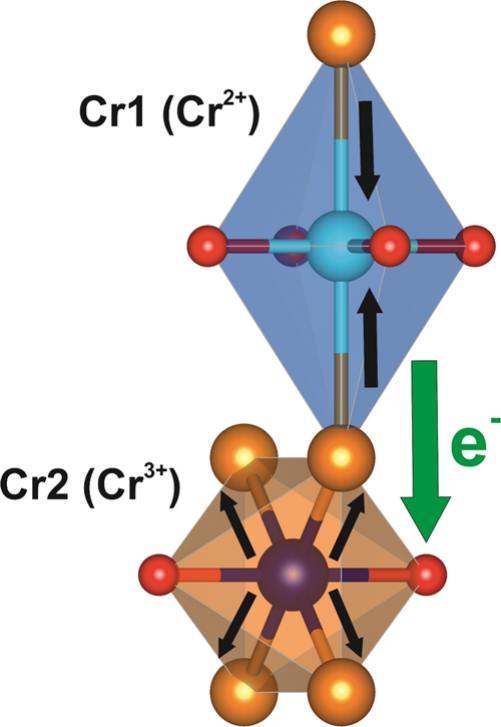
Schematic showing the small structural changes within the Cr polyhedra (as depicted by the black arrows) observed between 500 and 400 K as Sr_2_CrO_2_Cr_2_OAs_2_ is cooled. The changes in bond length (see Figure 18) are extremely small, and a possible driver for this transition would be a minuscule amount of electron transfer indicated by the green arrow (approx. 0.02 e^–^).

Upon initial analysis of the variable-temperature synchrotron XRPD data for Sr_2_CrO_2_Cr_2_OAs_2_ it was noticeable that the lattice parameters did not decrease in a linear fashion as the sample was cooled from 600 to 300 K. Instead, below 400 K, the rate by which the lattice parameters diminish increases for lattice parameter *a* but decreases for lattice parameter *c* perpendicular to the layers (Figure 18a). However, the decrease in unit cell volume adopts a linear trend (Figure 18b). We note that this deviation is extremely subtle and only readily evident because the data were collected with high resolution in temperature. The possibility that this is an experimental artifact was considered; however, the agreement factors for the sequential refinements do not show any anomalies. This observation prompted further investigation into changes in bond lengths and bond valence sums (BVS). The trends shown by these values in the region from 500 K down to 400 K differ from the trends exhibited in the 600–500 and 400–300 K regions. As the temperature is decreased, the Cr1 octahedra (CrO_4_As_2_) become marginally less distorted (Figure 18e) and the Cr2 octahedra (CrO_2_As_4_) become marginally more distorted (Figure 18g). In tandem with this structural change to the Cr polyhedra, the BVS value for Cr1 increases faster on cooling compared with the behavior in the 600–500 and 400–300 K regions, and the BVS for Cr2 becomes flat between these regions (Figure 18h). A plausible explanation could be that the Cr^3+^ ions in the Cr2 layers are being reduced by the Cr^2+^ in the Cr1 layers to a very small degree (of the order of 0.02 e^–^ according to the trends in the BVS). The high temperature magnetometry data in Figure 18i supports the idea that the driving force behind this structural change is likely to be electronic in origin as it shows a transition in the magnetic susceptibility of Sr_2_CrO_2_Cr_2_OAs_2_ between 500 and 400 K (i.e., exactly in the region of the structural change), which cannot be a signature of the magnetic ordering of the Cr2 and Cr1 layers as these have higher Néel temperatures of ∼600 and 530(10) K, respectively, i.e., the subtle structural change occurs below the temperature of magnetic long range ordering on both sublattices and is so small that we would not expect to observe any modulation of the ordered moments. We cannot completely rule out that the transition in the magnetometry is due to an impurity (although the small Sr_2_CrO_2_Cr_2_As_2_ impurity observed does not have transitions in this range), but this would not account for the structural observation. The nature of the structural distortion at each of the Cr sites is illustrated in Figure 19. Whether this structural change is due to a magnetostriction developing at the temperature where the ordered moments on the two independent Cr sublattices are both becoming saturated should also be considered.

High-temperature resistance experiments were attempted in this temperature range specifically to qualitatively test whether there was any observable electronic anomaly. The data suggest metallic behavior for this compound, due to the low resistance values and increase of resistance with increasing temperature, but did not show any transition in the 400–500 K region (Figure S6). The most likely explanations for the lack of an obvious transition are the relatively low sensitivity of the experimental setup and the extremely subtle nature of the structural change observed in the material. It is possible that the impurities in the sample also affected these measurements. We propose that further measurements of the structure, magnetism, and transport properties on single-crystal samples, perhaps backed up by computation to suggest whether the proposed internal redox process is plausible, would be required to shed further light on this subtle structural change.

## Conclusions

Two new phases, Sr_2_CrO_2_Cr_2_OAs_2_ and Sr_2_CrO_3_CrAs, have been synthesized in the bulk form after initially being highlighted or suggested by electron microscopy examination of the related Sr_2_CrO_2_Cr_2_As_2_ compound. Sr_2_CrO_2_Cr_2_OAs_2_ crystallizes in the *P*4/*mmm* space group and comprises two unique Cr sublattices—one containing Cr^2+^ in a CrO_4_As_2_ environment and the other Cr^3+^ in CrO_2_As_4_ coordination. Sr_2_CrO_3_CrAs also has Cr in two distinct layers, and in this case, CrO_5_ square-pyramids host Cr^3+^ cations and Cr^2+^ is in a CrAs_4_ tetrahedral coordination. This material adopts a structure with space group *P*4/*nmm*. The challenge that arises due to the competition between the formation of these two materials and Sr_2_CrO_2_Cr_2_As_2_—which only differ fairly slightly in their compositions (empirical formulae are Sr_2_Cr_3_As_2_O_3_, Sr_2_Cr_3_As_2_O_2_, and Sr_2_Cr_2_AsO_3_)—has been overcome through the use of synthetic optimization, and this has allowed sufficiently high levels of phase purity to be achieved for structural and magnetic analysis.

Sr_2_CrO_2_Cr_2_OAs_2_ exhibits long-range magnetic ordering on both Cr sublattices. The Cr^2+^ CrO_4_As_2_ moments align parallel to the *c* axis via antiferromagnetic Cr–O–Cr 180° superexchange interactions, whereas the Cr^3+^ CrO_2_As_4_ moments are best described as forming a checkerboard arrangement of antiferromagnetically coupled nearest-neighbor Cr centers, again with the moments directed along the *c*-direction. These observations can be rationalized by considering the various exchange interactions present and the preferential orientation of the moments with respect to the ligand field of the Cr centers. A Néel temperature of 530(10) K is evident for the three-dimensional long-range magnetic ordering on the Cr1 sublattice (CrO_2_ planes). This is significantly higher than the three-dimensional long-range ordering temperature for the Cr^2+^ moments in the similar layers in Sr_2_CrO_2_Cr_2_As_2_ and Ba_2_CrO_2_Cr_2_As_2_,^14,15^ presumably because in Sr_2_CrO_2_Cr_2_OAs_2_, adjacent layers of Cr1 moments are able to couple while in Sr_2_CrO_2_Cr_2_As_2_ and Ba_2_CrO_2_Cr_2_As_2_—where adjacent layers are related by body centering—there is no net coupling between adjacent layers. The long-range-ordered moment on the Cr2 sublattice dissipates at around 600 K. There is no clear feature in the magnetometry measurements of these antiferromagnetic ordering transitions, as is quite commonly the case for strongly two-dimensional systems.^48,49^ An electronic transition could be the driving force behind the very subtle structural distortions observed for the two Cr polyhedra between 500 and 400 K, accompanied by an anomaly in the magnetic susceptibility, but this subtle feature requires further investigation using single crystals.

In contrast, while NPD data show that the Cr^2+^ moments in the arsenide layers of Sr_2_CrO_3_CrAs are antiferromagnetically ordered over a long length scale with a saturated moment of 2.12(3) μ_B_, diffuse scattering below 40 K is consistent with only short-range antiferromagnetic ordering of the Cr^3+^ moments on the oxide layer in the *ab* plane. These moments do not appear to give rise to any sharp magnetic Bragg peaks.

## Supporting Information Available

Further diffractograms, refinement parameters, and magnetometry and conductivity data (PDF)
