# Supplementary material for: Structures and Magnetic Ordering in Layered Cr Oxide Arsenides Sr2CrO2Cr2OAs2 and Sr2CrO3CrAs
Source: Inorg Chem. 2022 Jul 27;61(31):12373–85. doi: 10.1021/acs.inorgchem.2c01773 (PMC9364410; doi:10.1021/acs.inorgchem.2c01773)
Supplement: Supplementary file 1 — ic2c01773_si_001.pdf [file ic2c01773_si_001.pdf]

Structures and Magnetic Ordering in Layered Cr Oxide Arsenides $Sr_2CrO_2Cr_2OAs_2$ and Sr_2CrO_3CrAs

Bradley C. Sheath,[a] Xiaoyu Xu,[a] Pascal Manuel,[b] Joke Hadermann,[c] Maria Batuk,[c] John O'Sullivan,[d] Ruy S. Bonilla,[d] and Simon J. Clarke[a,]*

[a]*Department of Chemistry, University of Oxford, Inorganic Chemistry Laboratory, South Parks Road, Oxford OX1 3QR, United Kingdom*

[b]*ISIS Facility, STFC Rutherford Appleton Laboratory, Harwell Oxford, Didcot OX11 0QX, United Kingdom*

[c]*Electron Microscopy for Materials Science (EMAT), University of Antwerp, Groenenborgerlaan 171, B-2020 Antwerp, Belgium*

[d]*Department of Materials, University of Oxford, Engineering and Technology Building, Parks Road, Oxford OX1 3PH, United Kingdom*

Supporting Information

email address: simon.clarke@chem.ox.ac.uk

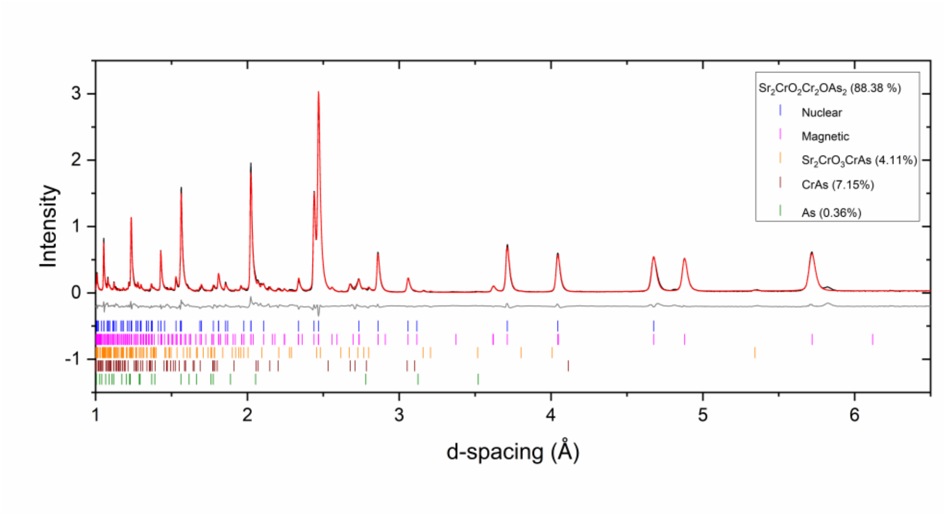

Figure S1. NPD pattern of $Sr_2CrO_2Cr_2OAs_2$ (combination of banks 3 and 8 with average $2\theta = 90°$) measured at 300 K on the WISH instrument at ISIS showing the observed (black), calculated (red) and difference (grey) curves. R_{wp}: 5.722%.

	$Sr_2CrO_2Cr_2OAs_2$ (RMM=529.1, *Z*=1)	
Diffractometer	WISH (ISIS)	I11 (MAC)
Wavelength (Å)	white beam TOF	0.826844
Radiation	Neutron	X-ray
d-space Range (Å)	0.55-48	1.2-18.9
Temperature (K)	300	300
Crystal System	Tetragonal	
Space Group	*P4/mmm* (123)	
a (Å)	4.04490(7)	4.040319(16)
c (Å)	9.35284(18)	9.33140(7)
V (Å^3)	153.024(6)	152.327(2)
z[Sr]	0.1838(2)	0.17664(8)
z[As]	0.3286(3)	0.32192(8)
Sr U_{11} (Å^2)	0.0084(7)	0.0024(2)
Sr U_{33} (Å^2)	0.0084(7)	0.0114(6)
Cr1 U_{11} (Å^2)	0.032(3)	0.0000(4)
Cr1 U_{33} (Å^2)	0.032(3)	0.0171(9)
Cr2 U_{11} (Å^2)	0.0073(12)	0.0002(3)
Cr2 U_{33} (Å^2)	0.0073(12)	0.0134(6)
O1 U_{iso} (Å^2)	0.0167(9)	0.0063(8)
O2 U_{iso}(Å^2)	0.0088(13)	0.0063(8)
As U_{11} (Å^2)	0.0116(8)	0.0000(3)
As U_{33} (Å^2)	0.0116(8)	0.0125(7)
Cr1–O (Å) [×4]	2.02245(4)	2.02016(1)
Cr1–As (Å) [×2]	3.0733(19)	3.0040(8)
Cr2–O (Å) [×2]	2.02245(4)	2.02016(1)
Cr2–As (Å) [×4]	2.5807(12)	2.6158(5)
χ^2	3.722x10^{-4}	4.901
R_p	4.941	5.939
R_{wp}	5.722	7.679

Table S1. Comparison of the refined parameters and selected bond lengths of $Sr_2CrO_2Cr_2OAs_2$. Refinement against the WISH (ISIS) data (Figure S1) was performed using isotropic displacement parameters for all atoms. Refinement against the I11 (MAC) data (Figure 4) was performed using isotropic displacement parameters for oxygen atoms only and both oxygen displacement parameters were set to refine to the same value. The low χ^2 value for the WISH refinement (Figure S1) is low as a consequence of the processing of the data from multiple detector banks. See note below for R-factor definitions.

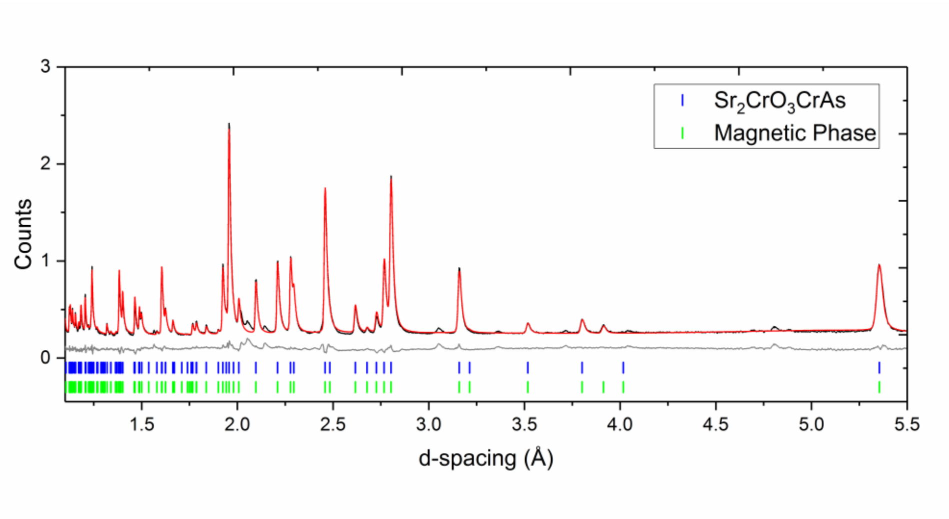

Figure S2. NPD pattern of Sr_2CrO_3CrAs (combination of banks 3 and 8 with average 2θ = 90°) measured at 293 K on the WISH instrument at ISIS showing the observed (black), calculated (red) and difference (grey) curves. R_{wp}: 4.499%.

	Sr_2CrO_3CrAs (RMM=402.2, *Z*=2)	
Diffractometer	WISH (ISIS)	I11 (MAC)
Wavelength (Å)	white beam TOF	0.825250
Radiation	Neutron	X-ray
d-space Range (Å)	0.55-48	1.2-18.9
Temperature (K)	293	298
Crystal System	Tetragonal	
Space Group	*P4/nmm* (129)	
a (Å)	3.91263(3)	3.909877(13)
c (Å)	16.06393(17)	16.05417(7)
V (Å^3)	245.917(4)	245.422(2)
z[Sr(1)]	0.19892(9)	0.19950(5)
z[Sr(2)]	0.41697(7)	0.41633(5)
z[Cr(1)]	0.31310(16)	0.31312(8)
z[As(1)]	0.09724(9)	0.09633(5)
z[O(1)]	0.29801(6)	0.29518(19)
z[O(2)]	0.43174(9)	0.4295(3)
Sr(1) U_{11} (Å^2)	0.0014(4)	0.0053(4)
Sr(1) U_{33} (Å^2)	0.0014(4)	0.0089(6)
Sr(2) U_{11} (Å^2)	0.0067(5)	0.0063(4)
Sr(2) U_{33} (Å^2)	0.0067(5)	0.0093(6)
Cr(1) U_{11} (Å^2)	0.0096(9)	0.0039(5)
Cr(1) U_{33} (Å^2)	0.0096(9)	0.0074(9)
Cr(2) U_{11} (Å^2)	0.0141(8)	0.0081(5)
Cr(2) U_{33}(Å^2)	0.0141(8)	0.0100(8)
As U_{11} (Å^2)	0.0063(5)	0.0075(4)
As U_{33} (Å^2)	0.0063(5)	0.0077(6)
O(1) U_{iso} (Å^2)	0.0077(4)	0.0130(10)
O(2) U_{iso} (Å^2)	0.0060(5)	0.101(13)
Cr(1)–O(1) (Å) [×4]	1.9713(4)	1.9760(5)
Cr(1)–O(2) (Å) [×1]	1.906(3)	1.868(5)
Cr(1)–As (Å) [×1]	3.468(3)	3.4804(16)
Cr(2)–As (Å) [×4]	2.5034(10)	2.4927(5)
As-Cr(2)-As (°) [×4]	112.91(3)	112.639(16)
As-Cr(2)-As (°) [×2]	102.79(6)	103.31(3)
χ^2	$3.497 x 10^{-4}$	22.171
R_p	3.289	4.847
R_{wp}	4.499	7.915

Table S2. Comparison of the refined parameters and selected bond lengths of Sr_2CrO_3CrAs. Refinement against the WISH (ISIS) data (Figure S2) was performed using isotropic displacement parameters for all atoms. Refinement against the I11 (MAC) data (Figure 5) was performed using isotropic displacement parameters for oxygen atoms only. The low χ^2 value for the WISH refinement (Figure S2) is low as a consequence of the processing of the data from multiple detector banks. See note below for R-factor definitions.

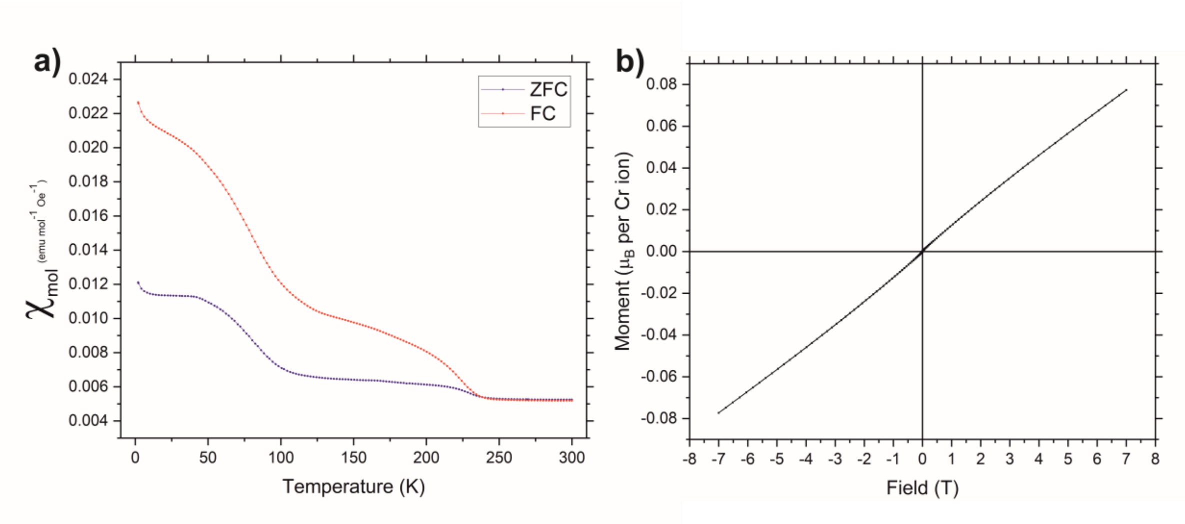

Figure S3. Magnetometry results for $Sr_2CrO_2Cr_2OAs_2$ showing **a)** low temperature Zero Field Cooled (ZFC) and Field Cooled (FC) curves measured in a field of 100 Oe and **b)** the hysteresis curve measured at 5 K.

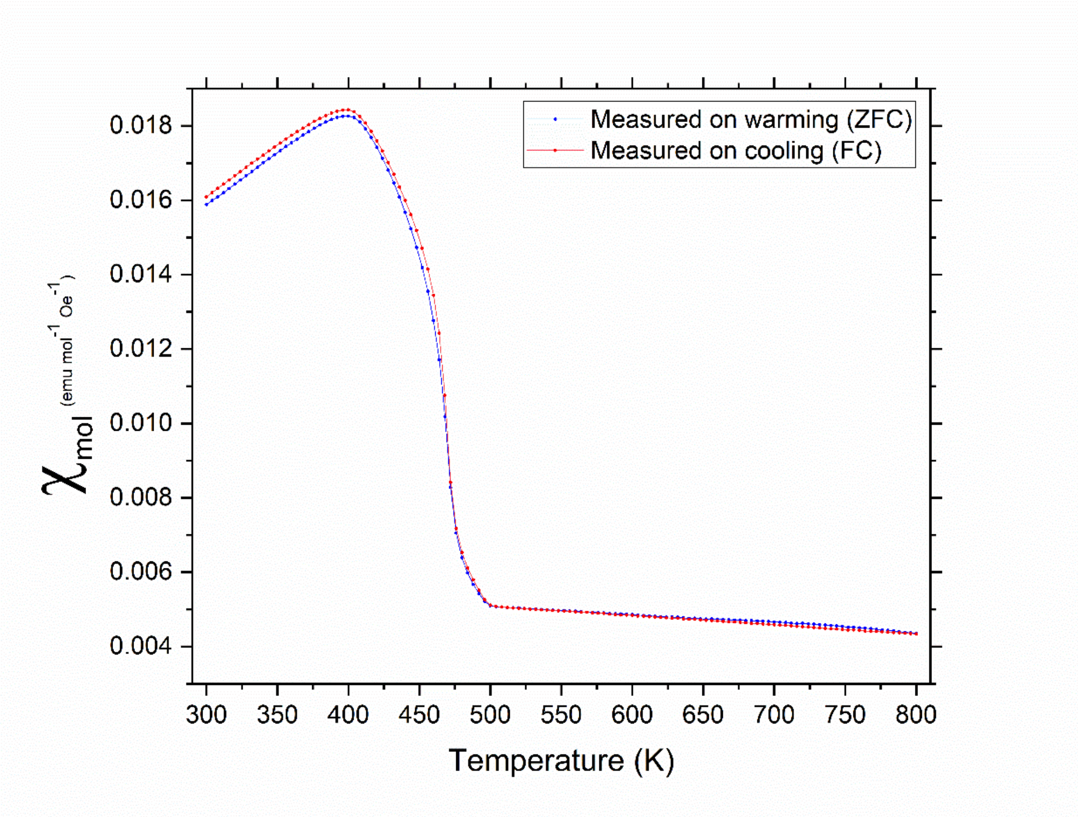

Figure S4. High temperature Zero Field Cooled (ZFC) and Field Cooled (FC) curves for $Sr_2CrO_2Cr_2OAs_2$ measured in a field of 100 Oe.

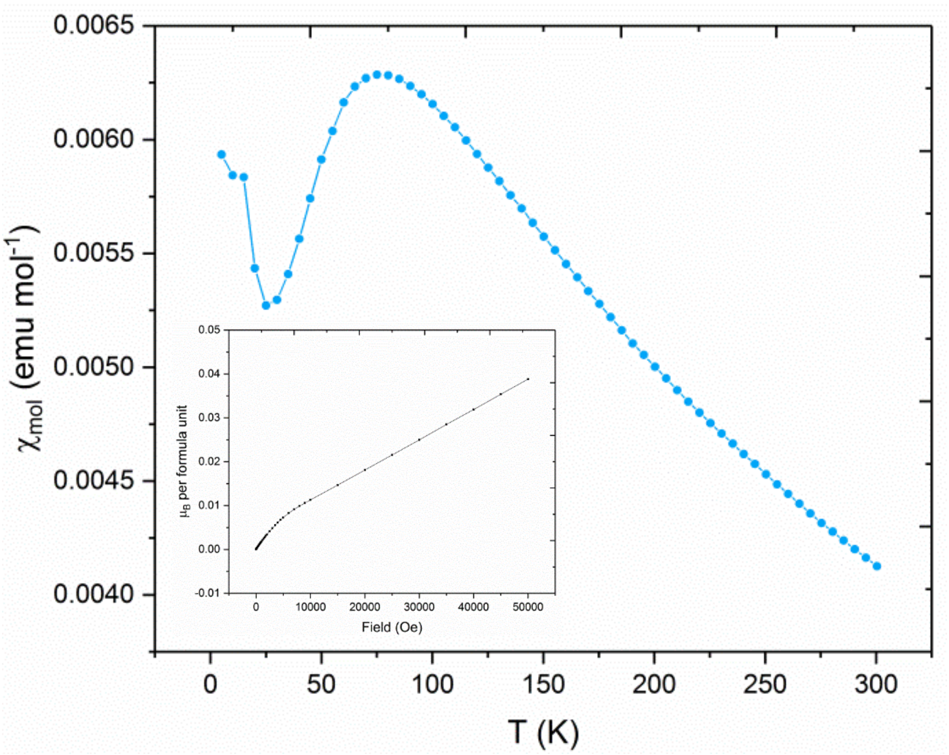

Figure S5. Field Cooled (FC) curve for Sr_2CrO_3CrAs measured using a 4T-3T subtraction. In this magnetic field range the minuscule ferromagnetic impurity causing the non-linear moment vs field curve (inset) is saturated, therefore the subtraction gives a more accurate picture of the intrinsic behaviour of Sr_2CrO_3CrAs.

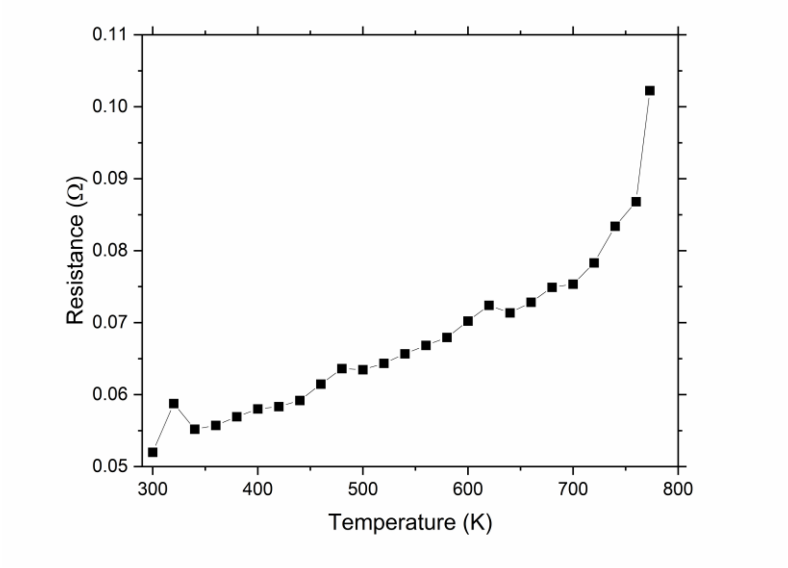

Figure S6. Resistance vs temperature curve for a sintered bar of the $Sr_2CrO_2Cr_2OAs_2$ material. There is no significant change in resistance between 400 K and 500 K.

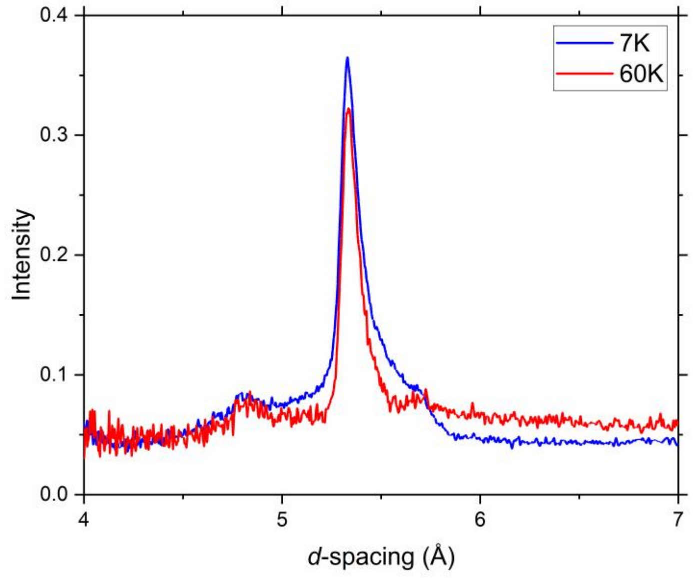

Figure S7. NPD patterns of Sr_2CrO_3CrAs (combination of banks 1 and 10 with average $2\theta = 26°$) at low temperatures measured on the WISH instrument at ISIS. It is evident that the background at the longer *d*-spacing values decreases and extra intensity arises where the magnetic correlations are (as highlighted in Fig. 17) as the sample is cooled from 60 K to 7 K. This is suggestive of some short-range magnetic order of the Cr^{3+} moments in the oxide layer.

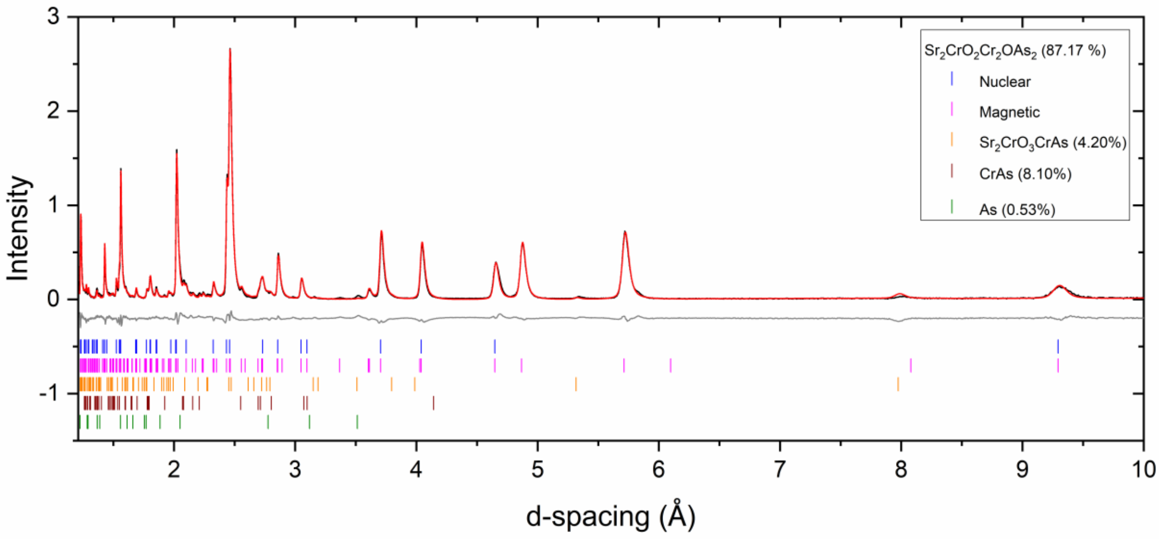

Fig. S8. NPD pattern of $Sr_2CrO_2Cr_2OAs_2$ (combination of banks 2 and 9 with average $2\theta = 58°$) measured at 10 K on the WISH instrument at ISIS showing the observed (black), calculated (red) and difference (grey) curves. R_{wp}: 5.751%.

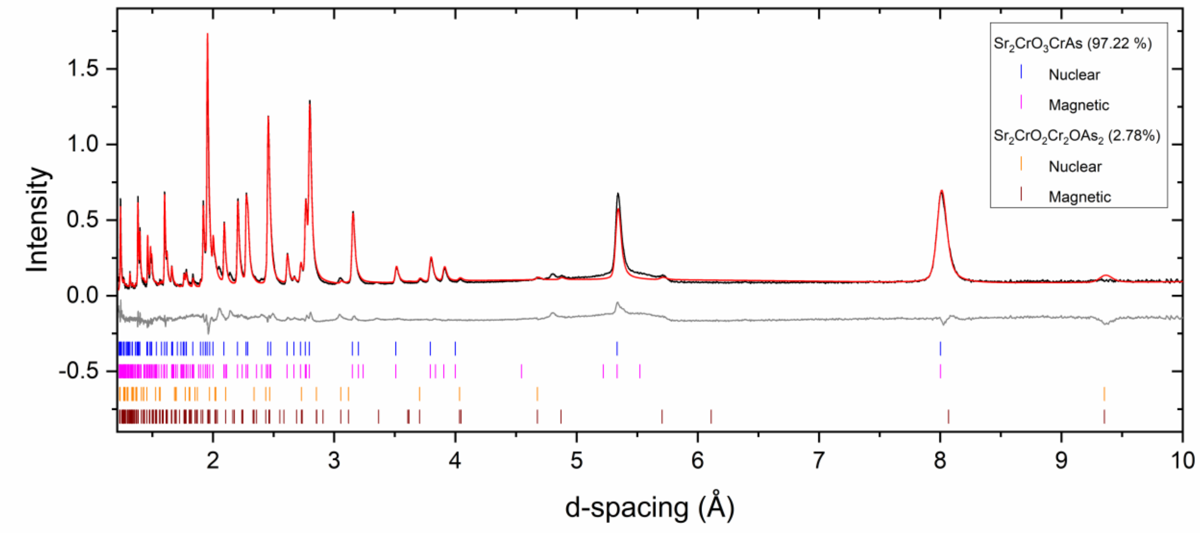

Fig. S9. NPD pattern of Sr_2CrO_3CrAs (combination of banks 2 and 9 with average $2\theta = 58°$) measured at 7 K on the WISH instrument at ISIS showing the observed (black), calculated (red) and difference (grey) curves. R_{wp}: 4.620%.

Note for Tables S1 and S2.

In the Rietveld refinement the function S_y is minimised

$$S_y = \sum_i w_i (y_i - y_{ci})^2$$

where y_i is the observed, and y_{ci} the calculated intensity at point *i* and w_i is the weighting factor, defined by $\frac{1}{y_i}$

The weighted profile *R* factor, R_{wp} is

$$R_{wp} = \sqrt{\frac{\sum_i w_i (y_i - y_{ci})^2}{\sum_i w_i y_i^2}}$$

The profile *R* factor, R_p is

$$R_p = \sqrt{\frac{\sum_i |y_i - y_{ci}|}{\sum_i y_i}}$$

The statistically expected *R* value, R_{exp}, in which all deviations of the calculated pattern from the observed pattern are due to statistical variations. R_{exp} is defined by:

$$R_{exp} = \sqrt{\frac{N_{obs} - N_{var}}{\sum_i w_i y_i^2}}$$

where N_{obs} and N_{var} are the number of observables and number of variables respectively.

A goodness of fit parameter, χ^2, is defined from the square of the ratio of R_{wp} and R_{exp}.
